# Supplementary material for: Cessation of smoking trial in the emergency department (CoSTED): protocol for a multicentre randomised controlled trial
Source: BMJ Open. 2023 Jan 18;13(1):e064585. doi: 10.1136/bmjopen-2022-064585 (PMC9853266; doi:10.1136/bmjopen-2022-064585)
Supplement: Author's manuscript [file bmjopen-2022-064585.draft_revisions.pdf]

BMJ Open is committed to open peer review. As part of this commitment we make the peer review history of every article we publish publicly available.

When an article is published we post the peer reviewers' comments and the authors' responses online. We also post the versions of the paper that were used during peer review. These are the versions that the peer review comments apply to.

The versions of the paper that follow are the versions that were submitted during the peer review process. They are not the versions of record or the final published versions. They should not be cited or distributed as the published version of this manuscript.

BMJ Open is an open access journal and the full, final, typeset and author-corrected version of record of the manuscript is available on our site with no access controls, subscription charges or pay-per-view fees (http://bmjopen.bmj.com).

If you have any questions on BMJ Open's open peer review process please email info.bmjopen@bmj.com

BMJ Open

Cessation of Smoking Trial in the Emergency Department (COSTED): protocol for a multicentre randomised controlled trial

Journal:	*BMJ Open*
Manuscript ID	bmjopen-2022-064585
Article Type:	Protocol
Date Submitted by the Author:	12-Jun-2022
Complete List of Authors:	Notley, Caitlin; University of East Anglia Faculty of Medicine and Health Sciences, Norwich Medical School<br>Clark, Lucy; University of East Anglia, Faculty of Medicine and Health Sciences<br>Belderson, P; University of East Anglia Faculty of Medicine and Health Sciences<br>Ward, Emma; University of East Anglia Faculty of Medicine and Health Sciences, Norwich Medical School<br>Clark, Allan; University of East Anglia Faculty of Medicine and Health Sciences, Norwich Medical School<br>Parrott, Steve; University of York<br>Agrawal, Sanjay; University of Leicester<br>Bloom, Ben; Barts Health NHS Trust, Emergency Department<br>Boyle, Adrian; Addenbrooke's Hospital, Emergency Medicine<br>Morris, Geraint; Homerton University Hospital NHS Foundation Trust, Department of Emergency Medicine<br>Gray, Alasdair; Royal Infirmary of Edinburgh, Department of Emergency Medicine<br>Coats, Tim; University of Leicester, Department of Cardiovascular Sciences<br>Man, Mei-See; University of East Anglia Faculty of Medicine and Health Sciences<br>Bauld, Linda; University of Edinburgh Division of Medical and Radiological Sciences, Usher Institute of Population Health Sciences and Informatics<br>Holland, Richard ; University of Leicester<br>Pope, Ian; Norfolk and Norwich University Hospitals NHS Foundation Trust, Department of Emergency Medicine
Keywords:	PUBLIC HEALTH, ACCIDENT & EMERGENCY MEDICINE, Protocols & guidelines < HEALTH SERVICES ADMINISTRATION & MANAGEMENT

SCHOLARONE™
Manuscripts

Cessation of Smoking Trial in the Emergency Department (COSTED): protocol for a multicentre randomised controlled trial

Caitlin Notley[1 *], Lucy Clark[2], Pippa Belderson[1], Emma Ward[1], Allan Clark[2], Steve Parrott[3], Sanjay Agrawal[4], Ben Bloom[5], Adrian Boyle[6], Geraint Morris[7], Alasdair Gray[8], Tim Coats[9], Mei-See Man[2], Linda Bauld[10], Richard Holland[11], Ian Pope[1, 12]

[*]Corresponding author. E mail: c.notley@uea.ac.uk; costed@uea.ac.uk Twitter: @CostedTrial

Author affiliations

[1] Faculty of Medicine and Health Sciences, University of East Anglia, Norwich, UK

[2] Norwich Clinical Trials Unit, Norwich Medical School, Faculty of Medicine and Health Sciences, University of East Anglia, Norwich, UK

[3] Department of Health Sciences, University of York, York, UK

[4] Institute of Lung Health, University of Leicester, Leicester, UK

[5] Department of Emergency Medicine, Royal London Hospital, Barts Health NHS Trust, London, UK

[6]Department of Emergency Medicine, Addenbrookes Hospital, Cambridge University Hospitals NHS Foundation Trust, Cambridge, UK

[7] Department of Emergency Medicine, Homerton University Hospital NHS Foundation Trust, London, UK

[8,10] Department of Emergency Medicine, Royal Infirmary of Edinburgh, Edinburgh, UK

[9] Department of Cardiovascular Sciences, University of Leicester, Leicester, UK

[10] The Usher Institute, College of Medicine and Veterinary Medicine, The University of Edinburgh, Edinburgh, UK

[11] Leicester Medical School, University of Leicester, Leicester, UK

[12] Department of Emergency Medicine, Norfolk and Norwich University Hospital, Norfolk and Norwich University Hospitals NHS Foundation Trust, Norwich, UK

For peer review only

ABSTRACT

Introduction:

Attendees of Emergency departments (ED) have a higher than expected prevalence of smoking. ED attendance may be a good opportunity to prompt positive behaviour change, even for smokers not currently motivated to quit. This study aims to determine whether an opportunist smoking cessation intervention delivered in the ED can help daily smokers attending the ED quit smoking and is cost-effective.

Methods and analysis:

A two-arm pragmatic, multicentred, parallel-group, individually randomised, controlled superiority trial with an internal pilot, economic evaluation and mixed methods process evaluation. The trial will compare ED-based brief smoking cessation advice, including provision of an e-cigarette and referral to local stop-smoking services (intervention) with the provision of contact details for local stop-smoking services (control). Target sample size is 972, recruiting across six National Health Service (NHS) EDs in England and Scotland. Outcomes will be collected at one, three and six months. The primary outcome at 6 months is Carbon Monoxide (CO) verified continuous smoking abstinence.

Ethics and dissemination:

The trial was approved by the South Central – Oxford B Research Committee (21/SC/0288). Dissemination will include the publication of outcomes, and the process and economic evaluations in peer reviewed journals. The findings will also be appropriately disseminated to relevant practice, policy and patient representative groups.

Trial registration number: NCT04854616; protocol version 3, 7th December 2021.

Strengths and limitations of this study

- This is the first study in the UK to assess the effectiveness of an emergency department delivered smoking cessation intervention using e-cigarettes opportunistically to aid smoking cessation in those not currently seeking smoking cessation advice
- The intervention development involved extensive patient and public involvement, particularly in the choice of e-cigarette
- The behavioural component of the intervention is person-centred, theory-based, scripted and delivered by advisors trained to deliver the support in the ED
- A mixed-methods process evaluation will assess the delivery, implementation, fidelity and contamination of the intervention through site observations and interviews with both staff and participants. An economic evaluation will establish the cost effectiveness of the intervention
- A potential limitation is that the control group received written information about local smoking cessation services rather than true ‘usual care’, which does not include written information

Word count: 3600

For peer review only

INTRODUCTION

Smoking cessation interventions usually target people who demonstrate initial motivation to quit. However, evidence suggests that 'unmotivated quitters' are also aware of the health risks of smoking and many of them are willing to consider quitting (1). Smoking rates are four times higher in the most disadvantaged populations compared to the most affluent (2), and those with mental health problems are far more likely to smoke (3,4). Both groups are also more likely to attend the Emergency Department (ED) (5). Our patient and public involvement (PPI) work, undertaken in 2019, showed that approximately 24% of ED attendees were current smokers, many more than in the wider population estimate of 15% in that same year (6). This makes an ED visit an ideal opportunity to screen for smoking status and deliver a smoking cessation intervention. There is good evidence that smokers in the ED are willing to consider quitting (7). Previous public health interventions in the ED have failed due to time pressures on ED staff delivering those interventions (8).

There is inconsistent evidence for smoking cessation interventions delivered in the ED (9), no UK evidence, and no studies have explored using e-cigarettes for smoking cessation support within the ED. E-cigarettes are now the most popular support method for smokers trying to stop (6) and there is increasing evidence that they are an effective way to quit (10). A Cochrane living review suggests e-cigarettes are twice as effective as nicotine replacement therapy when combined with behavioural support (11). However, initial start-up costs and lack of information may be barriers to first use (12). Providing an e-cigarette starter kit and brief advice, plus referral to support from local stop smoking services, may be a highly effective way of helping people to stop smoking, but has so far not been tested in populations recruited in an opportunistic setting that include unmotivated quitters and people who were not planning to access smoking cessation support.

This research will provide much needed evidence as to whether a smoking cessation intervention delivered in EDs using e-cigarettes is effective and improves treatment uptake in high prevalence disadvantaged population groups. This is key to meeting Government targets for populations in greatest need, including those in lower socioeconomic and hard to reach groups, and reducing health inequalities (13,14). This study will also provide evidence of the effectiveness of e-cigarettes for smoking cessation in smokers not planning a quit attempt.

For peer review only

AIMS & OBJECTIVES

The overarching aim of this trial is to determine if an individually tailored brief intervention that includes behavioural support, provision of an e-cigarette and referral to local smoking cessation services, provides an effective and cost-effective approach to supporting smoking abstinence and/or reduction in unmotivated quitters attending the ED.

The specific aims of the trial are to:

- run an internal pilot, with clear stop/go criteria, primarily to test recruitment systems.
- definitively test real-world effectiveness of an ED based brief, tailored, smoking cessation intervention in comparison with usual care, by comparing smoking abstinence at six month follow-up between trial groups.

- undertake a cost effectiveness analysis of the intervention in comparison with enhanced usual care from an NHS and personal social services (PSS) perspective.
- undertake an embedded mixed-methods process evaluation to assess intervention delivery, implementation, fidelity and contamination.

For peer review only

METHODS & ANALYSIS

This protocol is reported in accordance with the SPIRIT recommendations (15) and the TiDIER guidelines for intervention description (16).

Study design and setting

The COSTED trial is a pragmatic two-group, multicentred, randomised, controlled superiority trial with an internal pilot, economic evaluation and process evaluation. It will compare ED-based brief, individually tailored, smoking cessation advice including provision of an e-cigarette and referral to local stop-smoking services (intervention) with the provision of contact details for local stop-smoking services (control).

Recruitment to the trial will be over approximately 9 months from the 4th January 2022 in six NHS EDs pre-selected to maximise generalisability of findings based on being representative in deprivation, ethnicity, hospital size (including teaching and district general hospitals) and location (urban and rural). Additional sites will be included if recruitment to target is challenging.

Patient and public involvement

One member of the public is involved as named member of the trial steering group and a further three PPI representatives are involved in the trial management group and advisory group. Three PPI members contributed fully to intervention development by commenting, rating and assessing different e- cigarette starter kits, to enable informed choice of the intervention starter kit for the trial. PPI members have actively contributed to study design and materials and will continue to be consulted throughout the study and invited to contribute to dissemination.

Study population

Potential participants are approached in the ED. Participants are adults (≥ 18 years) who are daily tobacco smokers (≥1 cigarette or equivalent per day), verified by submitting an expired carbon monoxide (CO) breath test reading of ≥8 ppm. Smokers are ineligible if they require immediate medical treatment as defined by the treating clinician, are in police custody, report a known history of allergy to nicotine replacement products, are already using an e-cigarette daily, or do not have capacity to give informed consent for participation. For those requiring translation, the method already used for triage and clinical appointments in the ED is used, following local policies.

Potential participants admitted to hospital are not excluded, and if allocated to intervention, still receive it in the ED. They are given information on the local hospital smoking and e-cigarette policies.

Study procedures

Participant identification, approach, and consent

Potentially eligible participants are identified during their ED attendance by an ED researcher, or medical or nursing staff. Patients reporting smoking tobacco are provided with an information sheet (see figure 1). If eligible and interested, participants provide informed consent during their ED visit.

For peer review only

In the event of a person accompanying an eligible patient also meeting inclusion criteria and requesting to participate, the following procedure is followed: (1) if the patient did not consent to participate but the person accompanying them did consent, the person accompanying the patient is randomised, (2) if both the patient and the person accompanying them consented to participate, the patient is randomised and the accompanying person is allocated to receive the same treatment (intervention or control), but is not included as part of the trial population for the primary analysis.

Baseline assessment

Participants complete a baseline questionnaire face-to-face with a member of the local study team (table 1).

Table 1: Schedule of enrolment, assessments and interventions

	Enrolment	Allocation	Post-Allocation			
TIMEPOINT	At ED attendance	Following eligibility	Following allocation	1 and 3 month follow up		6 month follow up
**ENROLMENT**						
Eligibility screen	X					
Informed Consent	X					
Check eligibility including	X					X
Allocation		X				
ASSESSMENTS						
Demographics		X				
Self-reported smoking		X		X	X	X
Quit attempts		X				X
Time to relapse						X
Cigarette & tobacco use		X				X
Nicotine dependence		X				X
Motivation to stop smoking		X				X
E-cigarette use		X				X
Incidence of dry cough		X				X
Incidence of mouth or		X				X
Quality of life - EQ-5D-5L		X				X
Self-reported use of		X				X
Adverse events				X	X	X
Self-reported use of		X				X
INTERVENTIONS						
Smoking Cessation			X			
Referral to smoking			X			
E-cigarette given			X			
Signposting to NHS smoking			X			

Randomisation

Participants are individually randomised to intervention or control (1:1 ratio). Randomisation is by automated web-based system, computer generated by the UKCRC-registered Norwich Clinical Trials Unit (NCTU) using a blocked design, stratified by site.

For peer review only - http://bmjopen.bmj.com/site/about/guidelines.xhtml

Blinding

Due to the participatory nature of the intervention, it is impossible to blind participants or treating advisors to group allocation. Members of the trial management team will not be blinded to group allocation when assessing summary statistics for monitoring purposes throughout the trial.

Internal Pilot

The Independent Data Monitoring Committee (DMC) and Independent Trial Steering Committee (TSC) will scrutinise recruitment and protocol fidelity at three months into recruitment to establish continuation or stopping the trial at the pilot stage.

Follow up

1-month and 3 month Follow-Up

At one- and three-months post-randomisation, text messages are automatically generated by the study database and sent to all trial participants to ask about smoking status and any hospital admissions (to capture potential serious adverse events). If after two attempts there is no response, the central trial team follow-up by email, telephone, text or post.

6 Month Follow-up

At six months post randomisation, a link is automatically generated by the database and sent by text/email (depending on participant preference). The questionnaires consist of smoking status, time to relapse (if applicable), nicotine product use, e-cigarette use, use of healthcare and smoking cessation services in the past six months, self-reported dry cough and throat irritation, the Fagerstrom test for nicotine dependence (17) , the Motivation to Stop Smoking scale (MTSS) (17), quality of life (EQ-5D-5L) (18) and health service use (which captures potential serious adverse events). If participants do not respond the central trial team follows-up by email, telephone, text, or post.

For those who report smoking abstinence at six months, biochemical validation is requested by CO breath test by three potential mechanisms. Participants can either [1] return to the hospital they attended at baseline, [2] undertake remote collection with a CO monitor that is sent to them (an appointment will be made with the central or local team for a video-call to retrieve the reading), or [3] be visited at their home by a member of the local or central research team. On completing six month follow-up (including CO verification) randomised participants are reimbursed for their time with a £30 shopping voucher.

Trial Allocation Groups

Control

All trial participants are offered signposting to local NHS stop-smoking services through provision of written information. In the control arm this information is provided to the participant by the member of the research team taking consent; for the intervention group it forms part of the written information provided as part of the intervention.

Intervention

Those randomised to the intervention receive the COSTED intervention, which is an opportunistic smoking cessation intervention undertaken in the ED, comprising three elements: 1) brief smoking

For peer review only

cessation advice (up to 15 minutes), 2) the provision of an e-cigarette plus instruction in its use (up to 15 minutes), 3) referral to local stop-smoking services.

Following trial specific intervention training, the advice will be delivered individually (or with an accompanying person) by a smoking cessation advisor. Protocol driven advice addresses key aspects of the importance of switching away from tobacco smoking, tailored to the participant (e.g., discussing improved wound healing for patients attending with an open wound, or discussing increased risk of fractures for those who continue to smoke, for those attending with a fracture). This part of the intervention is a single session undertaken within the ED department, in a quiet area or in a separate room if available. Participants are provided with an e-cigarette starter kit (the DotPro starter kit, manufactured by Liberty Flights, an independent e cigarette manufacturer). which includes approximately two weeks supply of refill pods and training on its use. Participants are also offered referral to the local stop-smoking service who will provide routine follow up.

Those delivering the intervention, mainly nurses and health care assistants, undertook 2.5 days of standardised training and were provided with an intervention manual and videos . Training included online modules from the National Centre for Smoking Cessation (NCSCT) (NCSCT Training and Assessment Programme – core components, then speciality courses for certified practitioners, including modules on 'mental health and smoking cessation', 'pregnancy and smoking cessation', 'E-cigarettes: a guide for healthcare professionals', and 'very brief advice on second-hand smoke'). Following this, NCSCT Level 2 smoking cessation advisor training was delivered by SmokeFree Norfolk and bespoke training on use of e-cigarettes, which was initially delivered face-to-face and then video-recorded for future training. All those trained had the opportunity to undertake some role-play and/or shadow a trained advisor prior to delivering the intervention themselves. Methods to enhance and assess intervention fidelity are informed by the National Institutes of Health (NIH) Behaviour Change Consortium fidelity framework (19).

Sample size

The sample size was based on an estimated quit rate of 12.2% in the intervention group. This was based on a US trial of an ED smoking cessation intervention using a brief intervention, referral to smoking cessation services and nicotine replacement (19). A quit rate of 6.2% was used in the control group based on an average of three studies of unmotivated quitters who received either signposting or no intervention (19–21). This is also similar to the results of a recent Cochrane review of incentives reporting a quit rate of 7.2% amongst controls (22). A sample size using 90% power gives a required sample size of 972 (486 per group) at the 5% level of significance using a two-tailed test. No increase for dropout has been made as dropouts will be assumed to have returned to smoking as is standard in the smoking cessation literature (23).

Outcome measures

See Table 1

Primary outcome

The primary effectiveness outcome is self-reported continuous smoking abstinence, biochemically validated by CO monitoring at six months with a cut off of ≥8ppm (i.e., a reading of ≤7 will denote abstinence), according to the Russell standard (23).

Other outcome measures

Secondary outcomes measured at 6 months from randomisation:

1. 7-day point prevalence abstinence (i.e., current smoking status, self-report of having smoked no cigarettes (not even a puff) in the past seven days, biochemically validated by carbon monoxide monitoring with cut off of ≥8 ppm)
2. Number of quit attempts
3. Time to relapse (if applicable)
4. Number of cigarettes per day
5. Nicotine dependence (24)
6. Number of times using an e-cigarette per day
7. Incidence of self-reported dry cough or mouth or throat irritation(10)
8. Motivation to Stop Smoking (25)
9. Self-reported use of healthcare services in the last 6 months
10. Self-reported use of smoking cessation services in last 6 months
11. Quality of life (using the EQ-5D-5L)(18)
12. Self-reported smoking status and adverse events/reactions will be collected at 1 and 3 months.

Economic evaluation

The economic evaluation takes the form of an incremental cost-effectiveness analysis (CEA) from an NHS /PSS perspective on an ITT basis following NICE guidance (25). The costs comprise intervention/usual care costs and wider health care service costs. Intervention costs are prospectively recorded alongside the intervention delivery. A self-completion service use questionnaire is used to collect participants' utilisation of wider health care services at baseline and six month follow-up. A set of national average unit costs from secondary sources will be applied to derive a health care cost profile for each patient. We also collect participants' out-of-pocket expenditure in relation to smoking cessation to capture potential financial burdens for participants.

The economic evaluation will use both smoking abstinence at six months post-randomisation and Quality Adjusted Life Years (QALYs), derived from EQ-5D-5L (18), as outcomes.

The primary economic analysis is a within-trial CEA using total costs over the trial period and smoking abstinence at six months follow-up. As the effects of smoking cessation on health and health care utilisation are likely to occur beyond the trial period, to demonstrate the effects beyond the time horizon of the trial we will undertake model-based lifetime projections to estimate the long-term cost-effectiveness of the intervention. To reflect the full impact of smoking cessation on health, we will also estimate lifetime ICERs using model-based projections.

Process evaluation

A mixed methods process evaluation (26) will assess implementation and explore participant views on the intervention compared to usual care, contextual variation and potential contamination between the intervention and control group.

Participant interviews

Detailed qualitative data will be collected through semi-structured interviews with a purposive sample of both intervention and control group participants (total n=30) after completion of final follow-up. Participants will have been given the option at baseline to consent to being contacted about the qualitative interviews and formal consent will be sought.

For peer review only

Interviews will be undertaken face-to-face or remotely via telephone or video-conferencing and audio-recorded for transcription. The topic guide for intervention participants will enquire about views and experiences of the intervention, barriers and facilitators, and explore their longer-term perspective beyond the intervention period. For control participants the topic guide will explore experience of trail participation and access to usual care smoking cessation support, or any other form of support accessed.

Smoking cessation advisor interviews

At least one smoking cessation advisor from each of the six ED sites delivering the intervention will take part in a qualitative interview on completion of recruitment. Staff interviews will assess views and experiences of intervention delivery, giving an insider perspective on which parts of the intervention package are deemed to be most helpful, in which circumstances and with which participants. Barriers and facilitators to intervention delivery will be explored from the staff perspective, to aid interpretation of trial findings and triangulate with participants' qualitative data.

Interview data will be analysed thematically (26) , using a combination of inductive and deductive approaches to assess process elements of the trial. Initially all data will be analysed deductively, guided by the MRC guidance for complex interventions (27,28). Data will then be analysed inductively and more broadly. This will include critiquing the conceptual approach of CoSTED, guided by its logic model understanding any unintended consequences and reflections on the intervention from the participants' perspectives.

Site Observations

Observational data of the intervention delivery and ED setting will be collected at each of the six ED sites. A researcher will attend for at least three hours and take notes about the context, the interactions and conversation between patients approached for the trial and staff involved. A structured observation record sheet will be used for this purpose, prompting the researcher to observe the environment, interactions between key actors, the culture of the department, the flow of patients and any critical events that may impact on intervention delivery and implementation. The researcher will also broadly document details of the setting, including any smoking and vaping observed in and around the department. This will aid in assessing context of intervention delivery. A Site Profile Form, completed prior to site observations as part of the Site Initiation Visit (SIV), will capture information that will support the researcher observations. Observation notes will be coded thematically using QSR NVivo and triangulated with interview data.

Descriptive quantitative measures: fidelity, dose, and reach

Quantitative process measures such as recruitment and consent rates, retention and drop out, will be analysed to help provide an explanatory context for eventual trial outcomes. The smoking cessation advisor will complete a brief feedback form after each intervention contact to enable monitoring of fidelity (e.g., incorporation of motivational advice and device demonstration) and 'dose' (e.g., duration of consultation, interruptions to intervention delivery). The reach of the intervention will be evaluated using socio-demographic data collected for all participants.

For peer review only

Trial data handling

Data are collected and maintained in accordance with the current legal and regulatory requirements.

The redcap database and associated code have been developed by NCTU Data Management, in conjunction with the CoSTED trial team. The database software provides a number of features to help maintain data quality, including maintaining an audit trail, allowing custom validations on all data, allowing users to raise data query requests, and search facilities to identify validation failure/ missing data.

After completion of the trial, the database will be retained on the servers of NCTU for on-going analysis of secondary outcomes and held for five years in line with our data retention policy.

The identification, screening and enrolment logs, linking participant identifiable data to the pseudo-anonymised PIN, will be held centrally in REDCap to enable us to monitor screening progress and send out the SMS messages and emails. Screening forms will either be held in written form in a locked filing cabinet or electronically in a password protected form on hospital computers. After completion of the trial the identification, screening and enrolment logs will be stored securely by the sites for five years.

Qualitative interview data will be collected on an audio recorder, or by using computer recording via video conference, and immediately transferred to secure cloud storage (NCTU OneDrive) with access restricted to nominated members of the research team. Audio files will be transcribed verbatim by a member of UEA administrative staff or by an external transcription company. When transcribed, a researcher will assign each transcript a unique study specific number and/or code and remove all identifying information from the transcripts. The anonymised data will be used for analysis and stored securely for five years unless otherwise advised by NCTU.

Observational notes will also be assigned a unique identifying code and stored securely at UEA with access restricted to nominated members of the research team.

Data Analysis

A full statistical analysis plan was drafted during the trial delivery phase and will be reviewed and signed off by the independent DMC and TSC prior to database lock. The analyses will be reported in full and in accordance with the Consolidated Standards of Reporting Trials (CONSORT) guidelines(29). The main planned analyses are summarised below.

The comparison of outcomes between the treatment groups will be based on the intention-to-treat population. This will consist of all randomised individuals according to the group that they were allocated. For individuals who have missing data for the primary outcome it will be assumed that they are smoking. The primary outcome measure, smoking abstinence, will be compared between treatment groups using a binomial regression model adjusting for the factors stratified in the randomisation and any baseline factors that will be pre-specified by the TMG prior to database lock, and the decision will be made blind to treatment allocation. The effect size will be estimated as the relative risk (log link function in the binomial regression) and the risk difference (identity link function).

For peer review only

Secondary outcome measures will be analysed in a similar fashion, but the regression model will depend upon the outcome, specifically, binomial regression for binary outcomes, Poisson/Negative binomial for count outcomes and linear regression for continuous outcomes. If appropriate non-parametric methods will be used.

The pattern of missing data will be assessed, and multiple imputation methods will be used to assess for sensitivity of the results to assumptions around the missing data.

DISCUSSION

This pragmatic RCT aims to determine the effectiveness of an opportunistic smoking cessation intervention (provision of an e-cigarette, brief advice, and referral for stop smoking support) delivered to participants whilst in an Emergency Department. This trial is unique in that smoking cessation interventions in UK EDs have never, to our knowledge, been tested in a trial. This trial will provide valuable insights into a novel location for prompting positive behaviour change.

Current study status

The COSTED study started participant recruitment in January 2022. Data collection for the 6 month follow ups is expected to be completed in April 2023 and results are expected to be published in late 2023.

For peer review only

Acknowledgements We appreciate the support of the study sponsor, Norfolk and Norwich University Hospital NHS Foundation Trust. The study was led by researchers at the University of East Anglia (UEA) and managed by the Norwich Clinical Trials Unit (NCTU) at UEA. Our thanks go to all organisations involved in recruitment and in supporting delivery of the intervention, particularly local stop-smoking services. We would also like to thank PPI contributors who were critical in defining choice of e cigarette starter kit and shaping the intervention. We acknowledge the contribution of the researchers at sites who underwent training to deliver the intervention. We thank SmokeFree Norfolk for supporting the study by training all smoking advisors. Thanks also to our independent Trial Steering Committee and Data Monitoring committee members.

Contributors statement IP and CN conceived the idea for the study.CN, IP, EW and PB developed the intervention. LC is the trial manager and MM is the Senior Trail Manager. AC is the study statistician, SP is the Health Economist. SA, BB, AB, GM, AG, TC are site PIs, LB and RH provide academic expertise. All authors contributed to protocol writing and approved the final version.

Competing interests – None

Funding This study is funded by the National Institute for Health Research (NIHR) HTA programme Tobacco Cessation, Control and Harm Reduction (project reference 129438).

Disclaimer Neither the study sponsor or funder has a direct role in the study design; data collection, management, analysis and interpretation of data, report/manuscript writing or results dissemination. The views expressed are those of the author(s) and not necessarily those of the NIHR or the Department of Health and Social Care.

Data sharing statement Data underlying the main trial results will be available in a public, open access repository immediately following publication of the major findings of the study. Details of the repository and a persistent URL will be provided on publication of the main results. Study protocol, statistical analysis plan, informed consent form, and other documents will also be available.

REFERENCES

1. Chan SSC, Leung DYP, Abdullah ASM, Wong VT, Hedley AJ, Lam T-H. A randomized controlled trial of a smoking reduction plus nicotine replacement therapy intervention for smokers not willing to quit smoking. Addiction. 2011 Jun 1;106(6):1155–63.

2. Adult smoking habits in the UK - Office for National Statistics [Internet]. [cited 2019 Aug 7]. Available from: https://www.ons.gov.uk/peoplepopulationandcommunity/healthandsocialcare/healthandlifeexpectancies/bulletins/adultsmokinghabitsingreatbritain/2017#the-proportion-who-are-current-smokers-in-the-uk-its-constituent-countries-and-local-areas-2011-to-2017

3. Royal College of Physicians. Hiding in plain sight. Treating tobacco dependency in the NHS. A report by the Tobacco Advisory Group of the Royal College of Physicians. 2018.

4. Nicotine without smoke: Tobacco harm reduction [Internet]. RCP London. 2016 [cited 2018 Jul 31]. Available from: https://www.rcplondon.ac.uk/projects/outputs/nicotine-without-smoke-tobacco-harm-reduction-0

For peer review only

5. Keene J, Rodriguez J. Are mental health problems associated with use of Accident and Emergency and health-related harm? Eur J Public Health. 2007 Aug 1;17(4):387–93.

6. Latest Statistics - Smoking In England [Internet]. [cited 2020 Feb 3]. Available from: http://www.smokinginengland.info/latest-statistics/

7. Boudreaux ED, Baumann BM, Friedman K, Ziedonis DM. Smoking Stage of Change and Interest in an Emergency Department–based Intervention. Academic Emergency Medicine. 2005;12(3):211–8.

8. Drummond C, Deluca P, Coulton S, Bland M, Cassidy P, Crawford M, et al. The effectiveness of alcohol screening and brief intervention in emergency departments: a multicentre pragmatic cluster randomized controlled trial. PLoS One. 2014;9(6):e99463.

9. Lemhoefer C, Rabe GL, Wellmann J, Bernstein SL, Cheung KW, McCarthy WJ, et al. Emergency Department-Initiated Tobacco Control: Update of a Systematic Review and Meta-Analysis of Randomized Controlled Trials. Prev Chronic Dis. 2017;14:E89–E89.

10. Hajek P, Phillips-Waller A, Przulj D, Pesola F, Myers Smith K, Bisal N, et al. A Randomized Trial of E-Cigarettes versus Nicotine-Replacement Therapy. N Engl J Med. 2019 Jan 30;380(7):629–37.

11. Hartmann-Boyce J, McRobbie H, Butler AR, Lindson N, Bullen C, Begh R, et al. Electronic cigarettes for smoking cessation. Cochrane Database of Systematic Reviews [Internet]. 2021 [cited 2021 Jul 27];(4). Available from: https://www.cochranelibrary.com/cdsr/doi/10.1002/14651858.CD010216.pub5/full

12. English TM, Smith TB, Song X, Whitman MV. Barriers to electronic cigarette use. Public Health Nursing. 2018;35(5):363–8.

13. Department of Health and Social Care. Prevention is better than cure [Internet]. Available from: https://assets.publishing.service.gov.uk/government/uploads/system/uploads/attachment_data/file/753688/Prevention_is_better_than_cure_5-11.pdf

14. Health A on S and. Smoking Still Kills: Protecting Children, Reducing Inequalities. London: ASH; 2015.

15. Chan A-W, Tetzlaff JM, Altman DG, Laupacis A, Gøtzsche PC, Krleža-Jerić K, et al. SPIRIT 2013 statement: defining standard protocol items for clinical trials. Ann Intern Med. 2013 Feb 5;158(3):200–7.

16. Hoffmann TC, Glasziou PP, Boutron I, Milne R, Perera R, Moher D, et al. Better reporting of interventions: template for intervention description and replication (TIDieR) checklist and guide. BMJ. 2014 Mar 7;348:g1687.

17. Crittenden KS, Manfredi C, Lacey L, Warnecke R, Parsons J. Measuring readiness and motivation to quit smoking among women in public health clinics. Addictive Behaviors. 1994 Sep 1;19(5):497–507.

18. EQ-5D-5L – EQ-5D [Internet]. [cited 2020 Nov 8]. Available from: https://euroqol.org/eq-5d-instruments/eq-5d-5l-about/

For peer review only

19. Hennrikus D, Joseph AM, Lando HA. Effectiveness of a Smoking Cessation Program for Peripheral Artery Disease Patients: A Randomized Controlled Trial. Journal of Vascular Surgery. 2011 Jun 1;53(6):1752.

20. McFall M, Saxon AJ, Malte CA, Chow B, Bailey S, Baker DG, et al. Integrating Tobacco Cessation Into Mental Health Care for Posttraumatic Stress Disorder: A Randomized Controlled Trial. JAMA. 2010 Dec 8;304(22):2485–93.

21. Pieterse ME, Seydel ER, DeVries H, Mudde AN, Kok GJ. Effectiveness of a minimal contact smoking cessation program for Dutch general practitioners: a randomized controlled trial. Prev Med. 2001 Feb;32(2):182–90.

22. Notley C, Gentry S, Livingstone-Banks J, Bauld L, Perera R, Hartmann-Boyce J. Incentives for smoking cessation. Cochrane Database of Systematic Reviews [Internet]. 2019 [cited 2019 Aug 7];(7). Available from: https://www.cochranelibrary.com/cdsr/doi/10.1002/14651858.CD004307.pub6/full

23. West R. Assessing smoking cessation performance in NHS Stop Smoking Services: The Russell Standard (Clinical). 2005.

24. Heatherton TF, Kozlowski LT, Frecker RC, Fagerstrom K. The Fagerstrom Test for Nicotine Dependence: a revision of the Fagerstrom Tolerance Questionnaire. Br J Addict. 1991;1119–27.

25. 1 Introduction | Developing NICE guidelines: the manual | Guidance | NICE [Internet]. NICE; [cited 2022 Apr 7]. Available from: https://www.nice.org.uk/process/pmg20/chapter/introduction

26. Braun V, Clarke V. Using thematic analysis in psychology. Qualitative Research in Psychology. 2006 Jan 1;3(2):77–101.

27. Moore GF, Audrey S, Barker M, Bond L, Bonell C, Hardeman W, et al. Process evaluation of complex interventions: Medical Research Council guidance. BMJ. 2015 Mar 19;350:h1258.

28. Craig P, Dieppe P, Macintyre S, Michie S, Nazareth I, Petticrew M. Developing and evaluating complex interventions: the new Medical Research Council guidance. BMJ [Internet]. 2008 Sep 29;337. Available from: https://www.ncbi.nlm.nih.gov/pmc/articles/PMC2769032/

29. Tong A, Sainsbury P, Craig J. Consolidated criteria for reporting qualitative research (COREQ): a 32-item checklist for interviews and focus groups. Int J Qual Health Care. 2007 Dec 1;19(6):349–57.

For peer review only

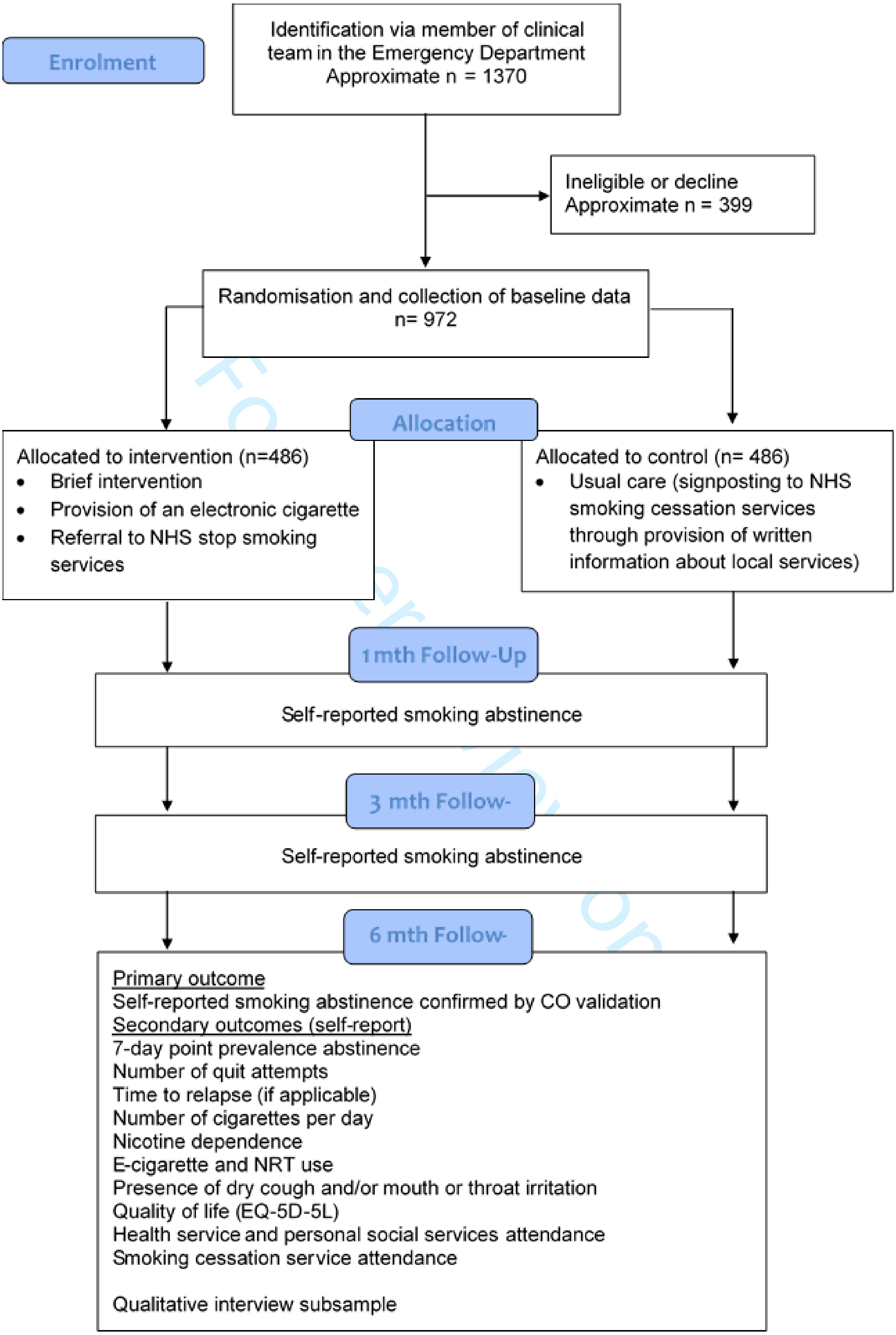

Figure 1: Participant Flow Diagram

For peer review only - http://bmjopen.bmj.com/site/about/guidelines.xhtml

TIDieR
Template for Intervention Description and Replication

The TIDieR (Template for Intervention Description and Replication) Checklist*:

Information to include when describing an intervention and the location of the information

Item number	Item	Where located ** Primary paper (page or appendix number)	Other † (details)
	**BRIEF NAME**		
**1.**	Provide the name or a phrase that describes the intervention.	__N/A_______	_______________
	**WHY**		
**2.**	Describe any rationale, theory, or goal of the elements essential to the intervention.	___page 3___	______________
	**WHAT**		
**3.**	Materials: Describe any physical or informational materials used in the intervention, including those provided to participants or used in intervention delivery or in training of intervention providers. Provide information on where the materials can be accessed (e.g. online appendix, URL).	_____________	______________
**4.**	Procedures: Describe each of the procedures, activities, and/or processes used in the intervention, including any enabling or support activities.	______	______________
	**WHO PROVIDED**		
**5.**	For each category of intervention provider (e.g. psychologist, nursing assistant), describe their expertise, background and any specific training given.	__page 7____	______________
	**HOW**		
**6.**	Describe the modes of delivery (e.g. face-to-face or by some other mechanism, such as internet or telephone) of the intervention and whether it was provided individually or in a group.	__page 7_____	______________
	**WHERE**		
**7.**	Describe the type(s) of location(s) where the intervention occurred, including any necessary infrastructure or relevant features.	___page 7___	______________

For peer review only - http://bmjopen.bmj.com/site/about/guidelines.xhtml

	**WHEN and HOW MUCH**		
**8.**	Describe the number of times the intervention was delivered and over what period of time including the number of sessions, their schedule, and their duration, intensity or dose.	__page 7____	______________
	**TAILORING**		
**9.**	If the intervention was planned to be personalised, titrated or adapted, then describe what, why, when, and how.	___page 7___	______________
	**MODIFICATIONS**		
**10.‡**	If the intervention was modified during the course of the study, describe the changes (what, why, when, and how).	_____N/A_________	______________
	**HOW WELL**		
**11.**	Planned: If intervention adherence or fidelity was assessed, describe how and by whom, and if any strategies were used to maintain or improve fidelity, describe them.	__ page 7 but not needed until outcome	______________
**12.‡**	Actual: If intervention adherence or fidelity was assessed, describe the extent to which the intervention was delivered as planned.	___N/A__________	______________

** **Authors** - use N/A if an item is not applicable for the intervention being described. **Reviewers** – use '?' if information about the element is not reported/not sufficiently reported.

† If the information is not provided in the primary paper, give details of where this information is available. This may include locations such as a published protocol or other published papers (provide citation details) or a website (provide the URL).

‡ If completing the TIDieR checklist for a protocol, these items are not relevant to the protocol and cannot be described until the study is complete.

* We strongly recommend using this checklist in conjunction with the TIDieR guide (see *BMJ* 2014;348:g1687) which contains an explanation and elaboration for each item.

* The focus of TIDieR is on reporting details of the intervention elements (and where relevant, comparison elements) of a study. Other elements and methodological features of studies are covered by other reporting statements and checklists and have not been duplicated as part of the TIDieR checklist. When a **randomised trial** is being reported, the TIDieR checklist should be used in conjunction with the CONSORT statement (see www.consort-statement.org) as an extension of **Item 5 of the CONSORT 2010 Statement.** When a **clinical trial protocol** is being reported, the TIDieR checklist should be used in conjunction with the SPIRIT statement as an extension of **Item 11 of the SPIRIT 2013**

For peer review only - http://bmjopen.bmj.com/site/about/guidelines.xhtml

Statement (see www.spirit-statement.org). For alternate study designs, TIDieR can be used in conjunction with the appropriate checklist for that study design (see www.equator-network.org).

For peer review only

For peer review only - http://bmjopen.bmj.com/site/about/guidelines.xhtml

BMJ Open

Cessation of Smoking Trial in the Emergency Department (CoSTED): protocol for a multicentre randomised controlled trial

Journal:	*BMJ Open*
Manuscript ID	bmjopen-2022-064585.R1
Article Type:	Protocol
Date Submitted by the Author:	03-Nov-2022
Complete List of Authors:	Notley, Caitlin; University of East Anglia Faculty of Medicine and Health Sciences, Norwich Medical School<br>Clark, Lucy; University of East Anglia, Faculty of Medicine and Health Sciences<br>Belderson, P; University of East Anglia Faculty of Medicine and Health Sciences<br>Ward, Emma; University of East Anglia Faculty of Medicine and Health Sciences, Norwich Medical School<br>Clark, Allan; University of East Anglia Faculty of Medicine and Health Sciences, Norwich Medical School<br>Parrott, Steve; University of York<br>Agrawal, Sanjay; University of Leicester<br>Bloom, Ben; Barts Health NHS Trust, Emergency Department<br>Boyle, Adrian; Addenbrooke's Hospital, Emergency Medicine<br>Morris, Geraint; Homerton University Hospital NHS Foundation Trust, Department of Emergency Medicine<br>Gray, Alasdair; Royal Infirmary of Edinburgh, Department of Emergency Medicine<br>Coats, Tim; University of Leicester, Department of Cardiovascular Sciences<br>Man, Mei-See; University of East Anglia Faculty of Medicine and Health Sciences<br>Bauld, Linda; University of Edinburgh Division of Medical and Radiological Sciences, Usher Institute of Population Health Sciences and Informatics<br>Holland, Richard ; University of Leicester<br>Pope, Ian; Norfolk and Norwich University Hospitals NHS Foundation Trust, Department of Emergency Medicine
**Primary Subject Heading**:	Emergency medicine
Secondary Subject Heading:	Public health, Addiction, Health services research
Keywords:	PUBLIC HEALTH, ACCIDENT & EMERGENCY MEDICINE, PREVENTIVE MEDICINE

SCHOLARONE™
Manuscripts

Cessation of Smoking Trial in the Emergency Department (CoSTED): protocol for a multicentre randomised controlled trial

Caitlin Notley[1 *], Lucy Clark[2], Pippa Belderson[1], Emma Ward[1], Allan Clark[2], Steve Parrott[3], Sanjay Agrawal[4], Ben Bloom[5], Adrian Boyle[6], Geraint Morris[7], Alasdair Gray[8], Tim Coats[9], Mei-See Man[2], Linda Bauld[10], Richard Holland[11], Ian Pope[1, 12]

[*]Corresponding author. E mail: c.notley@uea.ac.uk; costed@uea.ac.uk Twitter: @CostedTrial

Author affiliations

[1] Faculty of Medicine and Health Sciences, University of East Anglia, Norwich, UK

[2] Norwich Clinical Trials Unit, Norwich Medical School, Faculty of Medicine and Health Sciences, University of East Anglia, Norwich, UK

[3] Department of Health Sciences, University of York, York, UK

[4] Institute of Lung Health, University of Leicester, Leicester, UK

[5] Department of Emergency Medicine, Royal London Hospital, Barts Health NHS Trust, London, UK

[6]Department of Emergency Medicine, Addenbrookes Hospital, Cambridge University Hospitals NHS Foundation Trust, Cambridge, UK

[7] Department of Emergency Medicine, Homerton University Hospital NHS Foundation Trust, London, UK

[8,10] Department of Emergency Medicine, Royal Infirmary of Edinburgh, Edinburgh, UK

[9] Department of Cardiovascular Sciences, University of Leicester, Leicester, UK

[10] The Usher Institute, College of Medicine and Veterinary Medicine, The University of Edinburgh, Edinburgh, UK

[11] Leicester Medical School, University of Leicester, Leicester, UK

[12] Department of Emergency Medicine, Norfolk and Norwich University Hospital, Norfolk and Norwich University Hospitals NHS Foundation Trust, Norwich, UK

For peer review only

For peer review only - http://bmjopen.bmj.com/site/about/guidelines.xhtml

ABSTRACT

Introduction:

Attendees of Emergency departments (ED) have a higher than expected prevalence of smoking. ED attendance may be a good opportunity to prompt positive behaviour change, even for smokers not currently motivated to quit. This study aims to determine whether an opportunist smoking cessation intervention delivered in the ED can help daily smokers attending the ED quit smoking and is cost-effective.

Methods and analysis:

A two-arm pragmatic, multicentred, parallel-group, individually randomised, controlled superiority trial with an internal pilot, economic evaluation and mixed methods process evaluation. The trial will compare ED-based brief smoking cessation advice, including provision of an e-cigarette and referral to local stop-smoking services (intervention) with the provision of contact details for local stop-smoking services (control). Target sample size is 972, recruiting across six National Health Service (NHS) EDs in England and Scotland. Outcomes will be collected at one, three and six months. The primary outcome at 6 months is Carbon Monoxide (CO) verified continuous smoking abstinence.

Ethics and dissemination:

The trial was approved by the South Central – Oxford B Research Committee (21/SC/0288). Dissemination will include the publication of outcomes, and the process and economic evaluations in peer reviewed journals. The findings will also be appropriately disseminated to relevant practice, policy and patient representative groups.

Trial registration number: NCT04854616; protocol version 4.1.

Strengths and limitations of this study

- This is the first study in the UK to assess the effectiveness of an emergency department delivered smoking cessation intervention using e-cigarettes opportunistically to aid smoking cessation in those not currently seeking smoking cessation advice
- The intervention development involved extensive patient and public involvement, particularly in the choice of e-cigarette
- The behavioural component of the intervention is person-centred, theory-based, scripted and delivered by advisors trained to deliver the support in the ED
- A mixed-methods process evaluation will assess the delivery, implementation, fidelity and contamination of the intervention through site observations and interviews with both staff and participants. An economic evaluation will establish the cost effectiveness of the intervention
- A potential limitation is that the control group received written information about local smoking cessation services rather than true 'usual care', which does not include written information

Word count: 3600

For peer review only - http://bmjopen.bmj.com/site/about/guidelines.xhtml

INTRODUCTION

Smoking cessation interventions usually target people who demonstrate initial motivation to quit. However, evidence suggests that 'unmotivated quitters', i.e. people now actively seeking smoking cessation support at that time, are also aware of the health risks of smoking and many of them are willing to consider quitting (1). Smoking rates are four times higher in the most disadvantaged populations compared to the most affluent (2), and those with mental health problems are far more likely to smoke (3,4). Both groups are also more likely to attend the Emergency Department (ED) (5). Our patient and public involvement (PPI) work, undertaken in 2019, showed that approximately 24% of ED attendees were current smokers, many more than in the wider population estimate of 15% in that same year (6). This PPI work was undertaken at 4 EDs across the UK where a cross section of attendees were asked their smoking status. An ED visit is an ideal opportunity to screen for smoking status and deliver a smoking cessation intervention. There is good evidence that smokers in the ED are willing to consider quitting (7). Previous public health interventions in the ED have failed due to time pressures on ED staff delivering those interventions (8).

There is inconsistent evidence for smoking cessation interventions delivered in the ED (9), no UK evidence, and no studies have explored using e-cigarettes for smoking cessation support within the ED. E-cigarettes are now the most popular support method for smokers trying to stop (6) and there is increasing evidence that they are an effective way to quit (10). A Cochrane living review suggests e-cigarettes are twice as effective as nicotine replacement therapy when combined with behavioural support (11). However, initial start-up costs and lack of information may be barriers to first use (12). Providing an e-cigarette starter kit and brief advice, plus referral to support from local stop smoking services, may be a highly effective way of helping people to stop smoking, but has so far not been tested in populations recruited in an opportunistic setting that include unmotivated quitters and people who were not planning to access smoking cessation support.

This research will provide much needed evidence as to whether a smoking cessation intervention delivered in EDs using e-cigarettes is effective and improves treatment uptake in high prevalence disadvantaged population groups. This is key to meeting Government targets for populations in greatest need, including those in lower socioeconomic and hard to reach groups, and reducing health inequalities (13,14). This study will also provide evidence of the effectiveness of e-cigarettes for smoking cessation in smokers not planning a quit attempt.

AIMS & OBJECTIVES

The overarching aim of this trial is to determine if an individually tailored brief intervention that includes behavioural support, provision of an e-cigarette and referral to local smoking cessation services, provides an effective and cost-effective approach to supporting smoking abstinence and/or reduction in unmotivated quitters attending the ED.

The specific aims of the trial are to:

- run an internal pilot, with clear stop/go criteria, primarily to test recruitment systems.

For peer review only - http://bmjopen.bmj.com/site/about/guidelines.xhtml

- definitively test real-world effectiveness of an ED based brief, tailored, smoking cessation intervention in comparison with usual care, by comparing smoking abstinence at six month follow-up between trial groups.
- undertake a cost effectiveness analysis of the intervention in comparison with enhanced usual care from an NHS and personal social services (PSS) perspective.
- undertake an embedded mixed-methods process evaluation to assess intervention delivery, implementation, fidelity and contamination.

For peer review only

METHODS & ANALYSIS

This protocol is reported in accordance with the SPIRIT recommendations (15) and the TiDIER guidelines for intervention description (16).

Study design and setting

The CoSTED trial is a pragmatic two-group, multicentred, randomised, controlled superiority trial with an internal pilot, economic evaluation and process evaluation. It will compare ED-based brief, individually tailored, smoking cessation advice including provision of an e-cigarette and referral to local stop-smoking services (intervention) with the provision of contact details for local stop-smoking services (control).

Recruitment to the trial will be over approximately 9 months from the 4th January 2022 in six NHS EDs pre-selected to maximise generalisability of findings based on being representative in deprivation, ethnicity, hospital size (including teaching and district general hospitals) and location (urban and rural). Additional sites will be included if recruitment to target is challenging.

Patient and public involvement

One member of the public is involved as named member of the trial steering group and a further three PPI representatives are involved in the trial management group and advisory group. Three PPI members contributed fully to intervention development by commenting, rating and assessing different e- cigarette starter kits, to enable informed choice of the intervention starter kit for the trial. PPI members have actively contributed to study design and materials and will continue to be consulted throughout the study and invited to contribute to dissemination.

Study population

Potential participants are approached in the ED. Participants are adults (≥ 18 years) who are daily tobacco smokers (≥1 cigarette or equivalent per day), verified by submitting an expired carbon monoxide (CO) breath test reading of ≥8 ppm. Smokers are ineligible if they require immediate medical treatment as defined by the treating clinician, are in police custody, report a known history of allergy to nicotine replacement products, are already using an e-cigarette daily, or do not have capacity to give informed consent for participation. For those requiring translation, the method already used for triage and clinical appointments in the ED is used, following local policies.

Potential participants admitted to hospital are not excluded, and if allocated to intervention, still receive it in the ED. They are given information on the local hospital smoking and e-cigarette policies.

Study procedures

Participant identification, approach, and consent

Potentially eligible participants are identified during their ED attendance by an ED researcher, or medical or nursing staff. Patients reporting smoking tobacco are provided with an information sheet (see figure 1). If eligible and interested, participants provide informed consent during their ED visit.

For peer review only - http://bmjopen.bmj.com/site/about/guidelines.xhtml

In the event of a person accompanying an eligible patient also meeting inclusion criteria and requesting to participate, the following procedure is followed: (1) if the patient did not consent to participate but the person accompanying them did consent, the person accompanying the patient is randomised, (2) if both the patient and the person accompanying them consented to participate, the patient is randomised and the accompanying person is allocated to receive the same treatment (intervention or control), but is not included as part of the trial population for the primary analysis.

Baseline assessment

Participants complete a baseline questionnaire face-to-face with a member of the local study team (table 1).

Table 1: Schedule of enrolment, assessments and interventions

	Enrolment	Allocation	Post-Allocation			
**TIMEPOINT**	**At ED attendance**	**Following eligibility**	**Following allocation**	**1 and 3 month follow up**		**6 month follow up**
**ENROLMENT**						
Eligibility screen	X					
Informed Consent	X					
Check eligibility including CO monitor reading	X					X
Allocation		X				
ASSESSMENTS						
Demographics		X				
Self-reported smoking status		X		X	X	X
Quit attempts		X				X
Time to relapse						X
Cigarette & tobacco use		X				X
Nicotine dependence (Heatherton et		X				X
Motivation to stop smoking (Kotz et		X				X
E-cigarette use		X				X
Incidence of dry cough		X				X
Incidence of mouth or throat		X				X
Quality of life - EQ-5D-5L (EuroQol		X				X
Self-reported use of healthcare		X				X
Adverse events				X	X	X
Self-reported use of smoking		X				X
INTERVENTIONS						
Smoking Cessation Intervention			X			
Referral to smoking cessation service			X			
E-cigarette given			X			
Signposting to NHS smoking			X			

Randomisation

Participants are individually randomised to intervention or control (1:1 ratio). Randomisation is by automated web-based system, computer generated by the UKCRC-registered Norwich Clinical Trials Unit (NCTU) using a blocked design, stratified by site.

For peer review only - http://bmjopen.bmj.com/site/about/guidelines.xhtml

Blinding

Due to the participatory nature of the intervention, it is impossible to blind participants or treating advisors to group allocation. Members of the trial management team will not be blinded to group allocation when assessing summary statistics for monitoring purposes throughout the trial. Analysis of the primary and secondary outcomes will be undertaken by a statistician blind to group allocation.

Internal pilot

The Independent Data Monitoring Committee (DMC) and Independent Trial Steering Committee (TSC) will scrutinise recruitment and protocol fidelity at three months into recruitment to establish continuation or stopping the trial at the pilot stage.

Follow up

1 Month and 3 Month Follow-Up

At one- and three-months post-randomisation, text messages are automatically generated by the study database and sent to all trial participants to ask about smoking status and any hospital admissions (to capture potential serious adverse events). If after two attempts there is no response, the central trial team follow-up by email, telephone, text or post.

6 Month Follow-up

At six months post randomisation, a link is automatically generated by the database and sent by text/email (depending on participant preference). The questionnaires consist of smoking status, time to relapse (if applicable), nicotine product use, e-cigarette use, use of healthcare and smoking cessation services in the past six months, self-reported dry cough and throat irritation, the Fagerstrom test for nicotine dependence (17), the Motivation to Stop Smoking scale (MTSS) (18), quality of life (EQ-5D-5L) (19) and health service use (which captures potential serious adverse events). If participants do not respond the central trial team follows-up by email, telephone, text, or post.

For those who report smoking abstinence at six months, biochemical validation is requested by CO breath test by three potential mechanisms. Participants can either [1] return to the hospital they attended at baseline, [2] undertake remote collection with a CO monitor that is sent to them (an appointment will be made with the central or local team for a video-call to retrieve the reading), [3] be visited at their home by a member of the local or central research team, or [4] meet with a member of the local or central team at a mutually acceptable place. On completing six month follow-up (including CO verification) randomised participants are reimbursed for their time with a £30 shopping voucher.

Trial allocation groups

Control

All trial participants are offered signposting to local NHS stop-smoking services through provision of written information. In the control arm this information is provided to the participant by the member of the research team taking consent; for the intervention group it forms part of the written information provided as part of the intervention.

Intervention

For peer review only - http://bmjopen.bmj.com/site/about/guidelines.xhtml

Those randomised to the intervention receive the CoSTED intervention, which is an opportunistic smoking cessation intervention undertaken in the ED, comprising three elements: 1) brief theory driven ((20)smoking cessation advice (up to 15 minutes), 2) the provision of an e-cigarette plus instruction in its use (up to 15 minutes), 3) referral to local stop-smoking services.

Following trial specific intervention training, the advice will be delivered individually (or with an accompanying person) by a smoking cessation advisor. Protocol driven advice addresses key aspects of the importance of switching away from tobacco smoking, tailored to the participant (e.g., discussing improved wound healing for patients attending with an open wound, or discussing increased risk of fractures for those who continue to smoke, for those attending with a fracture). This part of the intervention is a single session undertaken within the ED department, in a quiet area or in a separate room if available. Participants are provided with an e-cigarette starter kit (the DotPro starter kit, manufactured by Liberty Flights, an independent e-cigarette manufacturer). which includes approximately two weeks supply of refill pods and training on its use. Participants are also offered referral to the local stop-smoking service who will provide routine follow up.

Those delivering the intervention, mainly nurses and health care assistants, undertook 2.5 days of standardised training and were provided with an intervention manual and videos . Training included online modules from the National Centre for Smoking Cessation (NCSCT) (NCSCT Training and Assessment Programme – core components, then speciality courses for certified practitioners, including modules on 'mental health and smoking cessation', 'pregnancy and smoking cessation', 'E-cigarettes: a guide for healthcare professionals', and 'very brief advice on second-hand smoke'). Following this, NCSCT Level 2 smoking cessation advisor training was delivered by SmokeFree Norfolk and bespoke training on use of e-cigarettes, which was initially delivered face-to-face and then video-recorded for future training. All those trained had the opportunity to undertake some role-play and/or shadow a trained advisor prior to delivering the intervention themselves. Methods to enhance and assess intervention fidelity are informed by the National Institutes of Health (NIH) Behaviour Change Consortium fidelity framework (19).

Sample size

The sample size was based on an estimated quit rate of 12.2% in the intervention group. This was based on a US trial of an ED smoking cessation intervention using a brief intervention, referral to smoking cessation services and nicotine replacement (21). A quit rate of 6.2% was used in the control group based on an average of three studies of unmotivated quitters who received either signposting or no intervention (22–24). This is also similar to the results of a recent Cochrane review of incentives reporting a quit rate of 7.2% amongst controls (25). A sample size using 90% power gives a required sample size of 972 (486 per group) at the 5% level of significance using a two-tailed test. No increase for dropout has been made as dropouts will be assumed to have returned to smoking as is standard in the smoking cessation literature (26).

Outcome measures

See Table 1

Primary outcome

The primary effectiveness outcome is self-reported continuous smoking abstinence, biochemically validated by CO monitoring at six months with a cut off of ≥8ppm (i.e., a reading of ≤7 will denote abstinence), according to the Russell standard (26).

Other outcome measures

Secondary outcomes measured at 6 months from randomisation:

For peer review only - http://bmjopen.bmj.com/site/about/guidelines.xhtml

1. 7-day point prevalence abstinence (i.e., current smoking status, self-report of having smoked no cigarettes (not even a puff) in the past seven days, biochemically validated by carbon monoxide monitoring with cut off of ≥8 ppm)
2. Number of quit attempts
3. Time to relapse (if applicable)
4. Number of cigarettes per day
5. Nicotine dependence (17)
6. Number of times using an e-cigarette per day
7. Incidence of self-reported dry cough or mouth or throat irritation(10)
8. Motivation to Stop Smoking (25)
9. Self-reported use of healthcare services in the last 6 months
10. Self-reported use of smoking cessation services in last 6 months
11. Quality of life (using the EQ-5D-5L)(19)
12. Self-reported smoking status and adverse events/reactions will be collected at 1 and 3 months.

Economic evaluation

The economic evaluation takes the form of an incremental cost-effectiveness analysis (CEA) from an NHS /PSS perspective on an 'intention to treat' (ITT) basis following NICE guidance (27). The costs comprise intervention/usual care costs and wider health care service costs. Intervention costs are prospectively recorded alongside the intervention delivery. A self-completion service use questionnaire is used to collect participants' utilisation of wider health care services at baseline and six month follow-up. A set of national average unit costs from secondary sources will be applied to derive a health care cost profile for each patient. We also collect participants' out-of-pocket expenditure in relation to smoking cessation to capture potential financial burdens for participants.

The economic evaluation will use both smoking abstinence at six months post-randomisation and Quality Adjusted Life Years (QALYs), derived from EQ-5D-5L (19), as outcomes.

The primary economic analysis is a within-trial CEA using total costs over the trial period and smoking abstinence at six months follow-up. As the effects of smoking cessation on health and health care utilisation are likely to occur beyond the trial period, to demonstrate the effects beyond the time horizon of the trial we will undertake model-based lifetime projections to estimate the long-term cost-effectiveness of the intervention. To reflect the full impact of smoking cessation on health, we will also estimate lifetime incremental cost-effectiveness ratios (ICERs) using model-based projections.

Process evaluation

A mixed methods process evaluation (26) will assess implementation and explore participant views on the intervention compared to usual care, contextual variation and potential contamination between the intervention and control group.

Participant interviews

Detailed qualitative data will be collected through semi-structured interviews with a purposive sample of both intervention and control group participants (total n=30) after completion of final follow-up. Participants will have been given the option at baseline to consent to being contacted about the qualitative interviews and formal consent will be sought.

For peer review only - http://bmjopen.bmj.com/site/about/guidelines.xhtml

Interviews will be undertaken face-to-face or remotely via telephone or video-conferencing and audio-recorded for transcription. The topic guide for intervention participants will enquire about views and experiences of the intervention, barriers and facilitators, and explore their longer-term perspective beyond the intervention period. For control participants the topic guide will explore experience of trial participation and access to usual care smoking cessation support, or any other form of support accessed.

Smoking cessation advisor interviews

At least one smoking cessation advisor from each of the six ED sites delivering the intervention will take part in a qualitative interview on completion of recruitment. Staff interviews will assess views and experiences of intervention delivery, giving an insider perspective on which parts of the intervention package are deemed to be most helpful, in which circumstances and with which participants. Barriers and facilitators to intervention delivery will be explored from the staff perspective, to aid interpretation of trial findings and triangulate with participants' qualitative data.

Interview data will be analysed thematically (28) , using a combination of inductive and deductive approaches to assess process elements of the trial. Initially all data will be analysed deductively, guided by the MRC guidance for complex interventions (29,30). Data will then be analysed inductively and more broadly. This will include critiquing the conceptual approach of CoSTED, guided by its logic model understanding any unintended consequences and reflections on the intervention from the participants' perspectives.

Site observations

Observational data of the intervention delivery and ED setting will be collected at each of the six ED sites. A researcher will attend for at least three hours and take notes about the context, the interactions and conversation between patients approached for the trial and staff involved. A structured observation record sheet will be used for this purpose, prompting the researcher to observe the environment, interactions between key actors, the culture of the department, the flow of patients and any critical events that may impact on intervention delivery and implementation. The researcher will also broadly document details of the setting, including any smoking and vaping observed in and around the department. This will aid in assessing context of intervention delivery. A Site Profile Form, completed prior to site observations as part of the Site Initiation Visit (SIV), will capture information that will support the researcher observations. Observation notes will be coded thematically using QSR NVivo and triangulated with interview data.

Descriptive quantitative measures: fidelity, dose, and reach

Quantitative process measures such as recruitment and consent rates, retention and drop out, will be analysed to help provide an explanatory context for eventual trial outcomes. The smoking cessation advisor will complete a brief feedback form after each intervention contact to enable monitoring of fidelity (e.g., incorporation of motivational advice and device demonstration) and 'dose' (e.g., duration of consultation, interruptions to intervention delivery). The reach of the intervention will be evaluated using socio-demographic data collected for all participants.

For peer review only - http://bmjopen.bmj.com/site/about/guidelines.xhtml

Trial data handling

Data are collected and maintained in accordance with the current legal and regulatory requirements.

The REDCap database and associated code have been developed by NCTU Data Management, in conjunction with the CoSTED trial team. The database software provides a number of features to help maintain data quality, including maintaining an audit trail, allowing custom validations on all data, allowing users to raise data query requests, and search facilities to identify validation failure/ missing data.

After completion of the trial, the database will be retained on the servers of NCTU for on-going analysis of secondary outcomes and held for five years in line with our data retention policy.

The identification, screening and enrolment logs, linking participant identifiable data to the pseudo-anonymised PIN, will be held centrally in REDCap to enable us to monitor screening progress and send out the SMS messages and emails. Screening forms will either be held in written form in a locked filing cabinet or electronically in a password protected form on hospital computers. After completion of the trial the identification, screening and enrolment logs will be stored securely by the sites for five years.

Qualitative interview data will be collected on an audio recorder, or by using computer recording via video conference, and immediately transferred to secure cloud storage (NCTU Sharepoint) with access restricted to nominated members of the research team. Audio files will be transcribed verbatim by a member of UEA administrative staff or by an external transcription company. When transcribed, a researcher will assign each transcript a unique study specific number and/or code and remove all identifying information from the transcripts. The anonymised data will be used for analysis and stored securely for five years unless otherwise advised by NCTU.

Observational notes will also be assigned a unique identifying code and stored securely at UEA with access restricted to nominated members of the research team.

Data analysis

A full statistical analysis plan was drafted during the trial delivery phase and will be reviewed and signed off by the independent DMC and TSC prior to database lock. The analyses will be reported in full and in accordance with the Consolidated Standards of Reporting Trials (CONSORT) guidelines (31). The main planned analyses are summarised below.

The comparison of outcomes between the treatment groups will be based on the ITT population. This will consist of all randomised individuals according to the group that they were allocated. For individuals who have missing data for the primary outcome it will be assumed that they are smoking. The primary outcome measure, smoking abstinence, will be compared between treatment groups using a binomial regression model adjusting for the factors stratified in the randomisation and any baseline factors that will be pre-specified by the Trial Management Group (TMG) prior to database lock, and the decision will be made blind to treatment allocation. The effect size will be estimated as the relative risk (log link function in the binomial regression) and the risk difference (identity link function).

For peer review only - http://bmjopen.bmj.com/site/about/guidelines.xhtml

Secondary outcome measures will be analysed in a similar fashion, but the regression model will depend upon the outcome, specifically, binomial regression for binary outcomes, Poisson/Negative binomial for count outcomes and linear regression for continuous outcomes. If appropriate non-parametric methods will be used.

The pattern of missing data will be assessed, and multiple imputation methods will be used to assess for sensitivity of the results to assumptions around the missing data.

DISCUSSION

This pragmatic RCT aims to determine the effectiveness of an opportunistic smoking cessation intervention (provision of an e-cigarette, brief advice, and referral for stop smoking support) delivered to participants whilst in an Emergency Department. This trial is unique in that smoking cessation interventions in UK EDs have never, to our knowledge, been tested in a trial. This trial will provide valuable insights into a novel location for prompting positive behaviour change.

Current study status

The CoSTED study started participant recruitment in January 2022. Data collection for the 6 month follow ups is expected to be completed in April 2023 and results are expected to be published in late 2023.

ETHICS AND DISSEMINATION:

The trial was approved by the South Central – Oxford B Research Committee (21/SC/0288). Dissemination will include the publication of outcomes, and the process and economic evaluations in peer reviewed journals. The findings will also be appropriately disseminated to relevant practice, policy and patient representative groups.

Acknowledgements We appreciate the support of the study sponsor, Norfolk and Norwich University Hospital NHS Foundation Trust (Norfolk and Norwich University Hospital Colney Lane Norwich NR4 7UY Tel: 01603 286286). The study was led by researchers at the University of East Anglia (UEA) and managed by the Norwich Clinical Trials Unit (NCTU) at UEA. Our thanks go to all organisations involved in recruitment and in supporting delivery of the intervention, particularly local stop-smoking services. We would also like to thank PPI contributors who were critical in defining choice of e cigarette starter kit and shaping the intervention. We acknowledge the contribution of the researchers at sites who underwent training to deliver the intervention. We thank SmokeFree Norfolk for supporting the study by training all smoking advisors. Thanks also to our independent Trial Steering Committee and Data Monitoring committee members.

Contributors statement IP and CN conceived the idea for the study.CN, IP, EW and PB developed the intervention. LC is the trial manager and MM is the Senior Trial Manager. AC is the study statistician, SP is the Health Economist. SA, BB, AB, GM, AG, TC are site PIs, LB and RH provide academic expertise. All authors contributed to protocol writing and approved the final version.

Competing interests – None

Funding This study is funded by the National Institute for Health Research (NIHR) HTA programme Tobacco Cessation, Control and Harm Reduction (project reference 129438).

For peer review only - http://bmjopen.bmj.com/site/about/guidelines.xhtml

Disclaimer Neither the study sponsor or funder has a direct role in the study design; data collection, management, analysis and interpretation of data, report/manuscript writing or results dissemination. The views expressed are those of the author(s) and not necessarily those of the NIHR or the Department of Health and Social Care.

Data sharing statement Data underlying the main trial results will be available in a public, open access repository immediately following publication of the major findings of the study. Details of the repository and a persistent URL will be provided on publication of the main results. Study protocol, statistical analysis plan, informed consent form, and other documents will also be available.

REFERENCES

1. Chan SSC, Leung DYP, Abdullah ASM, Wong VT, Hedley AJ, Lam TH. A randomized controlled trial of a smoking reduction plus nicotine replacement therapy intervention for smokers not willing to quit smoking. Addiction. 2011 Jun 1;106(6):1155–63.

2. Adult smoking habits in the UK - Office for National Statistics [Internet]. [cited 2019 Aug 7]. Available from: https://www.ons.gov.uk/peoplepopulationandcommunity/healthandsocialcare/healthandlifeexpectancies/bulletins/adultsmokinghabitsingreatbritain/2017#the-proportion-who-are-current-smokers-in-the-uk-its-constituent-countries-and-local-areas-2011-to-2017

3. Royal College of Physicians. Hiding in plain sight. Treating tobacco dependency in the NHS. A report by the Tobacco Advisory Group of the Royal College of Physicians. 2018.

4. Nicotine without smoke: Tobacco harm reduction [Internet]. RCP London. 2016 [cited 2018 Jul 31]. Available from: https://www.rcplondon.ac.uk/projects/outputs/nicotine-without-smoke-tobacco-harm-reduction-0

5. Keene J, Rodriguez J. Are mental health problems associated with use of Accident and Emergency and health-related harm? Eur J Public Health. 2007 Aug 1;17(4):387–93.

6. Latest Statistics - Smoking In England [Internet]. [cited 2020 Feb 3]. Available from: http://www.smokinginengland.info/latest-statistics/

7. Boudreaux ED, Baumann BM, Friedman K, Ziedonis DM. Smoking Stage of Change and Interest in an Emergency Department–based Intervention. Academic Emergency Medicine. 2005;12(3):211–8.

8. Drummond C, Deluca P, Coulton S, Bland M, Cassidy P, Crawford M, et al. The effectiveness of alcohol screening and brief intervention in emergency departments: a multicentre pragmatic cluster randomized controlled trial. PLoS One. 2014;9(6):e99463.

9. Lemhoefer C, Rabe GL, Wellmann J, Bernstein SL, Cheung KW, McCarthy WJ, et al. Emergency Department-Initiated Tobacco Control: Update of a Systematic Review and Meta-Analysis of Randomized Controlled Trials. Prev Chronic Dis. 2017;14:E89–E89.

10. Hajek P, Phillips-Waller A, Przulj D, Pesola F, Myers Smith K, Bisal N, et al. A Randomized Trial of E-Cigarettes versus Nicotine-Replacement Therapy. N Engl J Med. 2019 Jan 30;380(7):629–37.

For peer review only - http://bmjopen.bmj.com/site/about/guidelines.xhtml

11. Hartmann-Boyce J, McRobbie H, Butler AR, Lindson N, Bullen C, Begh R, et al. Electronic cigarettes for smoking cessation. Cochrane Database of Systematic Reviews [Internet]. 2021 [cited 2021 Jul 27];(4). Available from: https://www.cochranelibrary.com/cdsr/doi/10.1002/14651858.CD010216.pub5/full

12. English TM, Smith TB, Song X, Whitman MV. Barriers to electronic cigarette use. Public Health Nursing. 2018;35(5):363–8.

13. Department of Health and Social Care. Prevention is better than cure [Internet]. Available from: https://assets.publishing.service.gov.uk/government/uploads/system/uploads/attachment_data/file/753688/Prevention_is_better_than_cure_5-11.pdf

14. Health A on S and. Smoking Still Kills: Protecting Children, Reducing Inequalities. London: ASH; 2015.

15. Chan AW, Tetzlaff JM, Altman DG, Laupacis A, Gøtzsche PC, Krleža-Jerić K, et al. SPIRIT 2013 statement: defining standard protocol items for clinical trials. Ann Intern Med. 2013 Feb 5;158(3):200–7.

16. Hoffmann TC, Glasziou PP, Boutron I, Milne R, Perera R, Moher D, et al. Better reporting of interventions: template for intervention description and replication (TIDieR) checklist and guide. BMJ. 2014 Mar 7;348:g1687.

17. Heatherton TF, Kozlowski LT, Frecker RC, Fagerstrom K olov. The Fagerstrom Test for Nicotine Dependence: a revision of the Fagerstrom Tolerance Questionnaire. Br J Addict. 1991;1119–27.

18. Crittenden KS, Manfredi C, Lacey L, Warnecke R, Parsons J. Measuring readiness and motivation to quit smoking among women in public health clinics. Addictive Behaviors. 1994 Sep 1;19(5):497–507.

19. EQ-5D-5L – EQ-5D [Internet]. [cited 2020 Nov 8]. Available from: https://euroqol.org/eq-5d-instruments/eq-5d-5l-about/

20. Michie S, van Stralen MM, West R. The behaviour change wheel: A new method for characterising and designing behaviour change interventions. Implement Sci. 2011 Apr 23;6:42.

21. Bernstein SL, D'Onofrio G, Rosner J, O'Malley S, Makuch R, Busch S, et al. Successful Tobacco Dependence Treatment in Low-Income Emergency Department Patients: A Randomized Trial. Annals of Emergency Medicine. 2015 Aug;66(2):140–7.

22. Hennrikus D, Joseph AM, Lando HA. Effectiveness of a Smoking Cessation Program for Peripheral Artery Disease Patients: A Randomized Controlled Trial. Journal of Vascular Surgery. 2011 Jun 1;53(6):1752.

23. McFall M, Saxon AJ, Malte CA, Chow B, Bailey S, Baker DG, et al. Integrating Tobacco Cessation Into Mental Health Care for Posttraumatic Stress Disorder: A Randomized Controlled Trial. JAMA. 2010 Dec 8;304(22):2485–93.

24. Pieterse ME, Seydel ER, DeVries H, Mudde AN, Kok GJ. Effectiveness of a minimal contact smoking cessation program for Dutch general practitioners: a randomized controlled trial. Prev Med. 2001 Feb;32(2):182–90.

For peer review only

25. Notley C, Gentry S, Livingstone-Banks J, Bauld L, Perera R, Hartmann-Boyce J. Incentives for smoking cessation. Cochrane Database of Systematic Reviews [Internet]. 2019 [cited 2019 Aug 7];(7). Available from: https://www.cochranelibrary.com/cdsr/doi/10.1002/14651858.CD004307.pub6/full

26. West R. Assessing smoking cessation performance in NHS Stop Smoking Services: The Russell Standard (Clinical). 2005.

27. 1 Introduction | Developing NICE guidelines: the manual | Guidance | NICE [Internet]. NICE; [cited 2022 Apr 7]. Available from: https://www.nice.org.uk/process/pmg20/chapter/introduction

28. Braun V, Clarke V. Using thematic analysis in psychology. Qualitative Research in Psychology. 2006 Jan 1;3(2):77–101.

29. Moore GF, Audrey S, Barker M, Bond L, Bonell C, Hardeman W, et al. Process evaluation of complex interventions: Medical Research Council guidance. BMJ. 2015 Mar 19;350:h1258.

30. Craig P, Dieppe P, Macintyre S, Michie S, Nazareth I, Petticrew M. Developing and evaluating complex interventions: the new Medical Research Council guidance. BMJ [Internet]. 2008 Sep 29;337. Available from: https://www.ncbi.nlm.nih.gov/pmc/articles/PMC2769032/

31. Tong A, Sainsbury P, Craig J. Consolidated criteria for reporting qualitative research (COREQ): a 32-item checklist for interviews and focus groups. Int J Qual Health Care. 2007 Dec 1;19(6):349–57.

Figure legend:

Figure 1: Participant Flow Diagram

For peer review only

Figure 1: Participant Flow Diagram

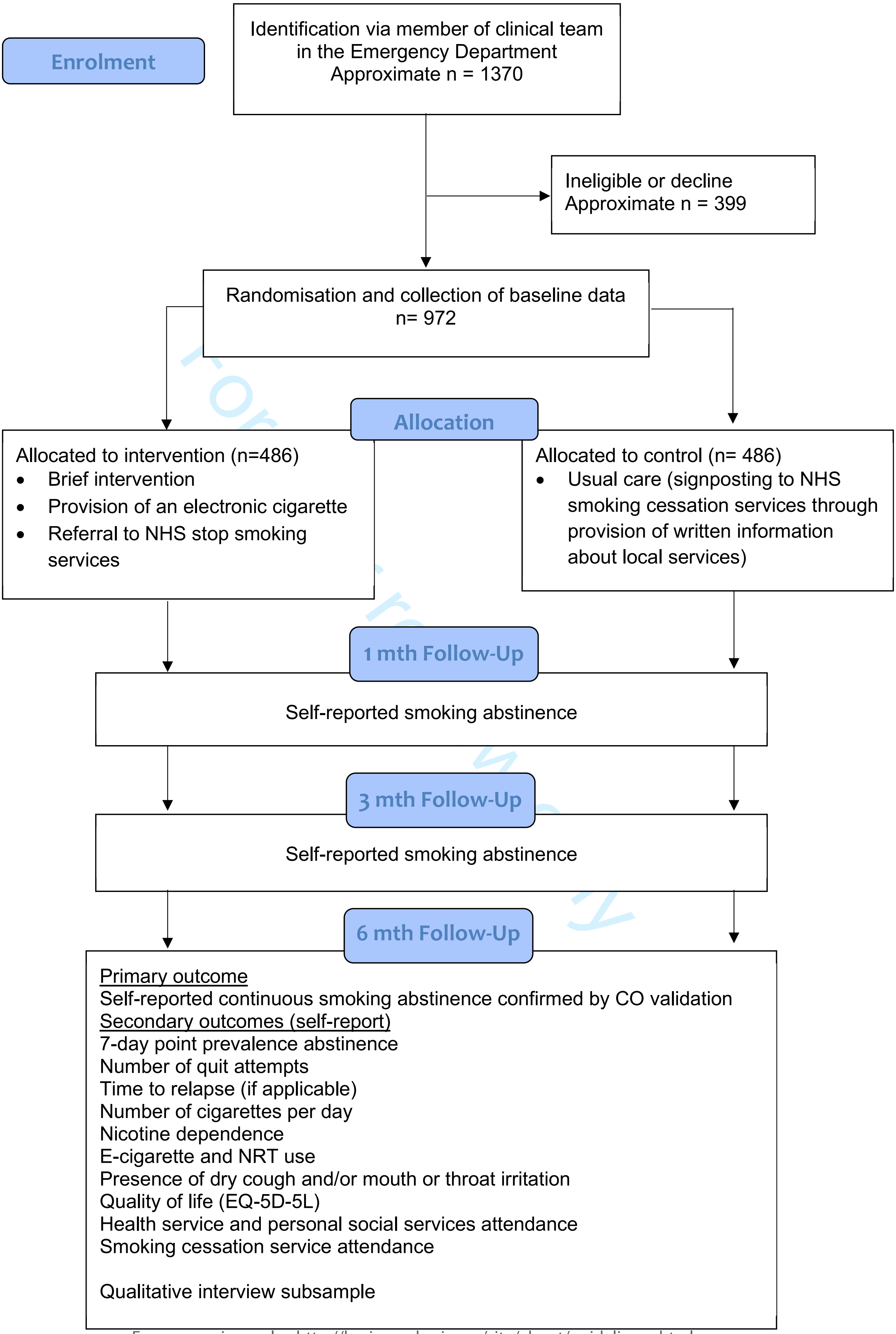

For peer review only

TIDieR
Template for Intervention Description and Replication

The TIDieR (Template for Intervention Description and Replication) Checklist*:

Information to include when describing an intervention and the location of the information

Item number	Item	Where located ** Primary paper (page or appendix number)	Other † (details)
	**BRIEF NAME**		
**1.**	Provide the name or a phrase that describes the intervention.	__N/A_______	_______________
	**WHY**		
**2.**	Describe any rationale, theory, or goal of the elements essential to the intervention.	___page 3___	______________
	**WHAT**		
**3.**	Materials: Describe any physical or informational materials used in the intervention, including those provided to participants or used in intervention delivery or in training of intervention providers. Provide information on where the materials can be accessed (e.g. online appendix, URL).	_____________	______________
**4.**	Procedures: Describe each of the procedures, activities, and/or processes used in the intervention, including any enabling or support activities.	______	______________
	**WHO PROVIDED**		
**5.**	For each category of intervention provider (e.g. psychologist, nursing assistant), describe their expertise, background and any specific training given.	__page 7____	______________
	**HOW**		
**6.**	Describe the modes of delivery (e.g. face-to-face or by some other mechanism, such as internet or telephone) of the intervention and whether it was provided individually or in a group.	__page 7_____	______________
	**WHERE**		
**7.**	Describe the type(s) of location(s) where the intervention occurred, including any necessary infrastructure or relevant features.	___page 7___	______________

For peer review only

For peer review only - http://bmjopen.bmj.com/site/about/guidelines.xhtml

	**WHEN and HOW MUCH**		
**8.**	Describe the number of times the intervention was delivered and over what period of time including the number of sessions, their schedule, and their duration, intensity or dose.	__page 7____	______________
	**TAILORING**		
**9.**	If the intervention was planned to be personalised, titrated or adapted, then describe what, why, when, and how.	___page 7___	______________
	**MODIFICATIONS**		
**10.‡**	If the intervention was modified during the course of the study, describe the changes (what, why, when, and how).	_____N/A_________	______________
	**HOW WELL**		
**11.**	Planned: If intervention adherence or fidelity was assessed, describe how and by whom, and if any strategies were used to maintain or improve fidelity, describe them.	__ page 7 but not needed until outcome	______________
**12.‡**	Actual: If intervention adherence or fidelity was assessed, describe the extent to which the intervention was delivered as planned.	___N/A__________	______________

** **Authors** - use N/A if an item is not applicable for the intervention being described. **Reviewers** – use '?' if information about the element is not reported/not sufficiently reported.

† If the information is not provided in the primary paper, give details of where this information is available. This may include locations such as a published protocol or other published papers (provide citation details) or a website (provide the URL).
‡ If completing the TIDieR checklist for a protocol, these items are not relevant to the protocol and cannot be described until the study is complete.

* We strongly recommend using this checklist in conjunction with the TIDieR guide (see *BMJ* 2014;348:g1687) which contains an explanation and elaboration for each item.

* The focus of TIDieR is on reporting details of the intervention elements (and where relevant, comparison elements) of a study. Other elements and methodological features of studies are covered by other reporting statements and checklists and have not been duplicated as part of the TIDieR checklist. When a **randomised trial** is being reported, the TIDieR checklist should be used in conjunction with the CONSORT statement (see www.consort-statement.org) as an extension of **Item 5 of the CONSORT 2010 Statement.** When a **clinical trial protocol** is being reported, the TIDieR checklist should be used in conjunction with the SPIRIT statement as an extension of **Item 11 of the SPIRIT 2013**

For peer review only - http://bmjopen.bmj.com/site/about/guidelines.xhtml
For peer review only

Statement (see www.spirit-statement.org). For alternate study designs, TIDieR can be used in conjunction with the appropriate checklist for that study design (see www.equator-network.org).

For peer review only

For peer review only - http://bmjopen.bmj.com/site/about/guidelines.xhtml

SPIRIT

STANDARD PROTOCOL ITEMS: RECOMMENDATIONS FOR INTERVENTIONAL TRIALS

SPIRIT 2013 Checklist: Recommended items to address in a clinical trial protocol and related documents*

Section/item	Item No	Description
**Administrative information**		
Title	1	Descriptive title identifying the study design, population, interventions, and, if applicable, trial acronym. Page 1.
Trial registration	2a	Trial identifier and registry name. If not yet registered, name of intended registry. Page 2.
	2b	All items from the World Health Organization Trial Registration Data Set N/A
Protocol version	3	Date and version identifier. Page 2.
Funding	4	Sources and types of financial, material, and other support. Page 12.
Roles and responsibilities	5a	Names, affiliations, and roles of protocol contributors. Page 1.
	5b	Name and contact information for the trial sponsor. Page 12.
	5c	Role of study sponsor and funders, if any, in study design; collection, management, analysis, and interpretation of data; writing of the report; and the decision to submit the report for publication, including whether they will have ultimate authority over any of these activities. Page 13.
	5d	Composition, roles, and responsibilities of the coordinating centre, steering committee, endpoint adjudication committee, data management team, and other individuals or groups overseeing the trial, if applicable (see Item 21a for data monitoring committee). Open Science framework.
**Introduction**		
Background and rationale	6a	Description of research question and justification for undertaking the trial, including summary of relevant studies (published and unpublished) examining benefits and harms for each intervention. Page 3
	6b	Explanation for choice of comparators. Page 7
Objectives	7	Specific objectives or hypotheses. Page 3-4

For peer review only

Trial design	8	Description of trial design including type of trial (eg, parallel group, crossover, factorial, single group), allocation ratio, and framework (eg, superiority, equivalence, noninferiority, exploratory). Pge 5.

Methods: Participants, interventions, and outcomes

Study setting	9	Description of study settings (eg, community clinic, academic hospital) and list of countries where data will be collected. Reference to where list of study sites can be obtained. Page 5.
Eligibility criteria	10	Inclusion and exclusion criteria for participants. If applicable, eligibility criteria for study centres and individuals who will perform the interventions (eg, surgeons, psychotherapists). Page 5.
Interventions	11a	Interventions for each group with sufficient detail to allow replication, including how and when they will be administered. Page 7-8
	11b	Criteria for discontinuing or modifying allocated interventions for a given trial participant (eg, drug dose change in response to harms, participant request, or improving/worsening disease) N/A as intervention delivered immediately following baseline assessment
	11c	Strategies to improve adherence to intervention protocols, and any procedures for monitoring adherence (eg, drug tablet return, laboratory tests) Page 10
	11d	Relevant concomitant care and interventions that are permitted or prohibited during the trial. N/A.
Outcomes	12	Primary, secondary, and other outcomes, including the specific measurement variable (eg, systolic blood pressure), analysis metric (eg, change from baseline, final value, time to event), method of aggregation (eg, median, proportion), and time point for each outcome. Explanation of the clinical relevance of chosen efficacy and harm outcomes is strongly recommended. Page 8 & Page 6 (table 1)
Participant timeline	13	Time schedule of enrolment, interventions (including any run-ins and washouts), assessments, and visits for participants. A schematic diagram is highly recommended (see Figure). Page 7.
Sample size	14	Estimated number of participants needed to achieve study objectives and how it was determined, including clinical and statistical assumptions supporting any sample size calculations. Page 8.
Recruitment	15	Strategies for achieving adequate participant enrolment to reach target sample size. Page 5/6.

Methods: Assignment of interventions (for controlled trials)

Allocation:

Sequence generation	16a	Method of generating the allocation sequence (eg, computer-generated random numbers), and list of any factors for stratification. To reduce predictability of a random sequence, details of any planned restriction (eg, blocking) should be provided in a separate document that is unavailable to those who enrol participants or assign interventions. Page 6.
Allocation concealment mechanism	16b	Mechanism of implementing the allocation sequence (eg, central telephone; sequentially numbered, opaque, sealed envelopes), describing any steps to conceal the sequence until interventions are assigned. Page 6.
Implementation	16c	Who will generate the allocation sequence, who will enrol participants, and who will assign participants to interventions. Page 6.
Blinding (masking)	17a	Who will be blinded after assignment to interventions (eg, trial participants, care providers, outcome assessors, data analysts), and how. Page 7.
	17b	If blinded, circumstances under which unblinding is permissible, and procedure for revealing a participant's allocated intervention during the trial. Page 7.
**Methods: Data collection, management, and analysis**		
Data collection methods	18a	Plans for assessment and collection of outcome, baseline, and other trial data, including any related processes to promote data quality (eg, duplicate measurements, training of assessors) and a description of study instruments (eg, questionnaires, laboratory tests) along with their reliability and validity, if known. Reference to where data collection forms can be found, if not in the protocol. Page 7.
	18b	Plans to promote participant retention and complete follow-up, including list of any outcome data to be collected for participants who discontinue or deviate from intervention protocols. Page 7.
Data management	19	Plans for data entry, coding, security, and storage, including any related processes to promote data quality (eg, double data entry; range checks for data values). Reference to where details of data management procedures can be found, if not in the protocol. Data sharing plan.
Statistical methods	20a	Statistical methods for analysing primary and secondary outcomes. Reference to where other details of the statistical analysis plan can be found, if not in the protocol. Page 11 & SAP.
	20b	Methods for any additional analyses (eg, subgroup and adjusted analyses). Page 11 & SAP.
	20c	Definition of analysis population relating to protocol non-adherence (eg, as randomised analysis), and any statistical methods to handle missing data (eg, multiple imputation). Page 11 & SAP.

Methods: Monitoring

Data monitoring	21a	Composition of data monitoring committee (DMC); summary of its role and reporting structure; statement of whether it is independent from the sponsor and competing interests; and reference to where further details about its charter can be found, if not in the protocol. Alternatively, an explanation of why a DMC is not needed. Open Science framework
	21b	Description of any interim analyses and stopping guidelines, including who will have access to these interim results and make the final decision to terminate the trial. Page 11 & SAP.
Harms	22	Plans for collecting, assessing, reporting, and managing solicited and spontaneously reported adverse events and other unintended effects of trial interventions or trial conduct. Page 7 and page 6 (table 1)
Auditing	23	Frequency and procedures for auditing trial conduct, if any, and whether the process will be independent from investigators and the sponsor. According to sponsor / funder request.

Ethics and dissemination

Research ethics approval	24	Plans for seeking research ethics committee/institutional review board (REC/IRB) approval. Page 12.
Protocol amendments	25	Plans for communicating important protocol modifications (eg, changes to eligibility criteria, outcomes, analyses) to relevant parties (eg, investigators, REC/IRBs, trial participants, trial registries, journals, regulators). Page 12,
Consent or assent	26a	Who will obtain informed consent or assent from potential trial participants or authorised surrogates, and how (see Item 32). Page 5/6
	26b	Additional consent provisions for collection and use of participant data and biological specimens in ancillary studies, if applicable. N/A.
Confidentiality	27	How personal information about potential and enrolled participants will be collected, shared, and maintained in order to protect confidentiality before, during, and after the trial. Page 11.
Declaration of interests	28	Financial and other competing interests for principal investigators for the overall trial and each study site. Page 12.
Access to data	29	Statement of who will have access to the final trial dataset, and disclosure of contractual agreements that limit such access for investigators. Page 11 and data management plan,
Ancillary and post-trial care	30	Provisions, if any, for ancillary and post-trial care, and for compensation to those who suffer harm from trial participation. N/A.

For peer review only - http://bmjopen.bmj.com/site/about/guidelines.xhtml

Dissemination policy	31a	Plans for investigators and sponsor to communicate trial results to participants, healthcare professionals, the public, and other relevant groups (eg, via publication, reporting in results databases, or other data sharing arrangements), including any publication restrictions. Page 12.
	31b	Authorship eligibility guidelines and any intended use of professional writers. N/A.
	31c	Plans, if any, for granting public access to the full protocol, participant-level dataset, and statistical code. Page 13.
**Appendices**		
Informed consent materials	32	Model consent form and other related documentation given to participants and authorised surrogates. Page 13 (to be made available on open science framework)
Biological specimens	33	Plans for collection, laboratory evaluation, and storage of biological specimens for genetic or molecular analysis in the current trial and for future use in ancillary studies, if applicable. N/A.

*It is strongly recommended that this checklist be read in conjunction with the SPIRIT 2013 Explanation & Elaboration for important clarification on the items. Amendments to the protocol should be tracked and dated. The SPIRIT checklist is copyrighted by the SPIRIT Group under the Creative Commons "Attribution-NonCommercial-NoDerivs 3.0 Unported" license.

For peer review only

BMJ Open

Cessation of Smoking Trial in the Emergency Department (CoSTED): protocol for a multicentre randomised controlled trial

Journal:	*BMJ Open*
Manuscript ID	bmjopen-2022-064585.R2
Article Type:	Protocol
Date Submitted by the Author:	17-Nov-2022
Complete List of Authors:	Notley, Caitlin; University of East Anglia Faculty of Medicine and Health Sciences, Norwich Medical School<br>Clark, Lucy; University of East Anglia, Faculty of Medicine and Health Sciences<br>Belderson, P; University of East Anglia Faculty of Medicine and Health Sciences<br>Ward, Emma; University of East Anglia Faculty of Medicine and Health Sciences, Norwich Medical School<br>Clark, Allan; University of East Anglia Faculty of Medicine and Health Sciences, Norwich Medical School<br>Parrott, Steve; University of York<br>Agrawal, Sanjay; University of Leicester<br>Bloom, Ben; Barts Health NHS Trust, Emergency Department<br>Boyle, Adrian; Addenbrooke's Hospital, Emergency Medicine<br>Morris, Geraint; Homerton University Hospital NHS Foundation Trust, Department of Emergency Medicine<br>Gray, Alasdair; Royal Infirmary of Edinburgh, Department of Emergency Medicine<br>Coats, Tim; University of Leicester, Department of Cardiovascular Sciences<br>Man, Mei-See; University of East Anglia Faculty of Medicine and Health Sciences<br>Bauld, Linda; University of Edinburgh Division of Medical and Radiological Sciences, Usher Institute of Population Health Sciences and Informatics<br>Holland, Richard ; University of Leicester<br>Pope, Ian; Norfolk and Norwich University Hospitals NHS Foundation Trust, Department of Emergency Medicine
**Primary Subject Heading**:	Emergency medicine
Secondary Subject Heading:	Public health, Addiction, Health services research
Keywords:	PUBLIC HEALTH, ACCIDENT & EMERGENCY MEDICINE, PREVENTIVE MEDICINE

Cessation of Smoking Trial in the Emergency Department (CoSTED): protocol for a multicentre randomised controlled trial

Caitlin Notley[1 *], Lucy Clark[2], Pippa Belderson[1], Emma Ward[1], Allan Clark[2], Steve Parrott[3], Sanjay Agrawal[4], Ben Bloom[5], Adrian Boyle[6], Geraint Morris[7], Alasdair Gray[8], Tim Coats[9], Mei-See Man[2], Linda Bauld[10], Richard Holland[11], Ian Pope[1, 12]

[*]Corresponding author. E mail: c.notley@uea.ac.uk; costed@uea.ac.uk Twitter: @CostedTrial

Author affiliations

[1] Faculty of Medicine and Health Sciences, University of East Anglia, Norwich, UK

[2] Norwich Clinical Trials Unit, Norwich Medical School, Faculty of Medicine and Health Sciences, University of East Anglia, Norwich, UK

[3] Department of Health Sciences, University of York, York, UK

[4] Institute of Lung Health, University of Leicester, Leicester, UK

[5] Department of Emergency Medicine, Royal London Hospital, Barts Health NHS Trust, London, UK

[6]Department of Emergency Medicine, Addenbrookes Hospital, Cambridge University Hospitals NHS Foundation Trust, Cambridge, UK

[7] Department of Emergency Medicine, Homerton University Hospital NHS Foundation Trust, London, UK

[8,10] Department of Emergency Medicine, Royal Infirmary of Edinburgh, Edinburgh, UK

[9] Department of Cardiovascular Sciences, University of Leicester, Leicester, UK

[10] The Usher Institute, College of Medicine and Veterinary Medicine, The University of Edinburgh, Edinburgh, UK

[11] Leicester Medical School, University of Leicester, Leicester, UK

[12] Department of Emergency Medicine, Norfolk and Norwich University Hospital, Norfolk and Norwich University Hospitals NHS Foundation Trust, Norwich, UK

For peer review only - http://bmjopen.bmj.com/site/about/guidelines.xhtml

ABSTRACT

Introduction:

Attendees of Emergency departments (ED) have a higher than expected prevalence of smoking. ED attendance may be a good opportunity to prompt positive behaviour change, even for smokers not currently motivated to quit. This study aims to determine whether an opportunist smoking cessation intervention delivered in the ED can help daily smokers attending the ED quit smoking and is cost-effective.

Methods and analysis:

A two-arm pragmatic, multicentred, parallel-group, individually randomised, controlled superiority trial with an internal pilot, economic evaluation and mixed methods process evaluation. The trial will compare ED-based brief smoking cessation advice, including provision of an e-cigarette and referral to local stop-smoking services (intervention) with the provision of contact details for local stop-smoking services (control). Target sample size is 972, recruiting across six National Health Service (NHS) EDs in England and Scotland. Outcomes will be collected at one, three and six months. The primary outcome at 6 months is Carbon Monoxide (CO) verified continuous smoking abstinence.

Ethics and dissemination:

The trial was approved by the South Central – Oxford B Research Committee (21/SC/0288). Dissemination will include the publication of outcomes, and the process and economic evaluations in peer reviewed journals. The findings will also be appropriately disseminated to relevant practice, policy and patient representative groups.

Trial registration number: NCT04854616; protocol version 4.1.

Strengths and limitations of this study

- This is the first study in the UK to assess the effectiveness of an emergency department delivered smoking cessation intervention using e-cigarettes opportunistically to aid smoking cessation in those not currently seeking smoking cessation advice
- The intervention development involved extensive patient and public involvement, particularly in the choice of e-cigarette
- The behavioural component of the intervention is person-centred, theory-based, scripted and delivered by advisors trained to deliver the support in the ED
- A mixed-methods process evaluation will assess the delivery, implementation, fidelity and contamination of the intervention through site observations and interviews with both staff and participants. An economic evaluation will establish the cost effectiveness of the intervention
- A potential limitation is that the control group received written information about local smoking cessation services rather than true 'usual care', which does not include written information

Word count: 3600

For peer review only - http://bmjopen.bmj.com/site/about/guidelines.xhtml

INTRODUCTION

Smoking cessation interventions usually target people who demonstrate initial motivation to quit. However, evidence suggests that 'unmotivated quitters', i.e. people not actively seeking smoking cessation support at that time, are also aware of the health risks of smoking and many of them are willing to consider quitting (1). Smoking rates are four times higher in the most disadvantaged populations compared to the most affluent (2), and those with mental health problems are far more likely to smoke (3,4). Both groups are also more likely to attend the Emergency Department (ED) (5). Our patient and public involvement (PPI) work, undertaken in 2019, showed that approximately 24% of ED attendees were current smokers, many more than in the wider population estimate of 15% in that same year (6). This PPI work was undertaken at 4 EDs across the UK where a cross section of attendees were asked their smoking status. An ED visit is an ideal opportunity to screen for smoking status and deliver a smoking cessation intervention. There is good evidence that smokers in the ED are willing to consider quitting (7). Previous public health interventions in the ED have failed due to time pressures on ED staff delivering those interventions (8).

There is inconsistent evidence for smoking cessation interventions delivered in the ED (9), no UK evidence, and no studies have explored using e-cigarettes for smoking cessation support within the ED. E-cigarettes are now the most popular support method for smokers trying to stop (6) and there is increasing evidence that they are an effective way to quit (10). A Cochrane living review suggests e-cigarettes are twice as effective as nicotine replacement therapy when combined with behavioural support (11). However, initial start-up costs and lack of information may be barriers to first use (12). Providing an e-cigarette starter kit and brief advice, plus referral to support from local stop smoking services, may be a highly effective way of helping people to stop smoking, but has so far not been tested in populations recruited in an opportunistic setting that include unmotivated quitters and people who were not planning to access smoking cessation support.

This research will provide much needed evidence as to whether a smoking cessation intervention delivered in EDs using e-cigarettes is effective and improves treatment uptake in high prevalence disadvantaged population groups. This is key to meeting Government targets for populations in greatest need, including those in lower socioeconomic and hard to reach groups, and reducing health inequalities (13,14). This study will also provide evidence of the effectiveness of e-cigarettes for smoking cessation in smokers not planning a quit attempt.

AIMS & OBJECTIVES

The overarching aim of this trial is to determine if an individually tailored brief intervention that includes behavioural support, provision of an e-cigarette and referral to local smoking cessation services, provides an effective and cost-effective approach to supporting smoking abstinence and/or reduction in unmotivated quitters attending the ED.

The specific aims of the trial are to:

- run an internal pilot, with clear stop/go criteria, primarily to test recruitment systems.

For peer review only - http://bmjopen.bmj.com/site/about/guidelines.xhtml

- definitively test real-world effectiveness of an ED based brief, tailored, smoking cessation intervention in comparison with usual care, by comparing smoking abstinence at six month follow-up between trial groups.
- undertake a cost effectiveness analysis of the intervention in comparison with enhanced usual care from an NHS and personal social services (PSS) perspective.
- undertake an embedded mixed-methods process evaluation to assess intervention delivery, implementation, fidelity and contamination.

For peer review only

METHODS & ANALYSIS

This protocol is reported in accordance with the SPIRIT recommendations (15) and the TiDIER guidelines for intervention description (16).

Study design and setting

The CoSTED trial is a pragmatic two-group, multicentred, randomised, controlled superiority trial with an internal pilot, economic evaluation and process evaluation. It will compare ED-based brief, individually tailored, smoking cessation advice including provision of an e-cigarette and referral to local stop-smoking services (intervention) with the provision of contact details for local stop-smoking services (control).

Recruitment to the trial will be over approximately 9 months from the 4th January 2022 in six NHS EDs pre-selected to maximise generalisability of findings based on being representative in deprivation, ethnicity, hospital size (including teaching and district general hospitals) and location (urban and rural). Additional sites will be included if recruitment to target is challenging.

Patient and public involvement

One member of the public is involved as named member of the trial steering group and a further three PPI representatives are involved in the trial management group and advisory group. Three PPI members contributed fully to intervention development by commenting, rating and assessing different e- cigarette starter kits, to enable informed choice of the intervention starter kit for the trial. PPI members have actively contributed to study design and materials and will continue to be consulted throughout the study and invited to contribute to dissemination.

Study population

Potential participants are approached in the ED. Participants are adults (≥ 18 years) who are daily tobacco smokers (≥1 cigarette or equivalent per day), verified by submitting an expired carbon monoxide (CO) breath test reading of ≥8 ppm. Smokers are ineligible if they require immediate medical treatment as defined by the treating clinician, are in police custody, report a known history of allergy to nicotine replacement products, are already using an e-cigarette daily, or do not have capacity to give informed consent for participation. For those requiring translation, the method already used for triage and clinical appointments in the ED is used, following local policies.

Potential participants admitted to hospital are not excluded, and if allocated to intervention, still receive it in the ED. They are given information on the local hospital smoking and e-cigarette policies.

Study procedures

Participant identification, approach, and consent

Potentially eligible participants are identified during their ED attendance by an ED researcher, or medical or nursing staff. Patients reporting smoking tobacco are provided with an information sheet (see figure 1). If eligible and interested, participants provide informed consent during their ED visit.

For peer review only - http://bmjopen.bmj.com/site/about/guidelines.xhtml

In the event of a person accompanying an eligible patient also meeting inclusion criteria and requesting to participate, the following procedure is followed: (1) if the patient did not consent to participate but the person accompanying them did consent, the person accompanying the patient is randomised, (2) if both the patient and the person accompanying them consented to participate, the patient is randomised and the accompanying person is allocated to receive the same treatment (intervention or control), but is not included as part of the trial population for the primary analysis.

Baseline assessment

Participants complete a baseline questionnaire face-to-face with a member of the local study team (table 1).

Table 1: Schedule of enrolment, assessments and interventions

	Enrolment	Allocation	Post-Allocation			
TIMEPOINT	At ED attendance	Following eligibility	Following allocation	1 and 3 month follow up		6 month follow up
**ENROLMENT**						
Eligibility screen	X					
Informed Consent	X					
Check eligibility including CO monitor reading	X					X
Allocation		X				
ASSESSMENTS						
Demographics		X				
Self-reported smoking status		X		X	X	X
Quit attempts		X				X
Time to relapse						X
Cigarette & tobacco use		X				X
Nicotine dependence (Heatherton et		X				X
Motivation to stop smoking (Kotz et		X				X
E-cigarette use		X				X
Incidence of dry cough		X				X
Incidence of mouth or throat		X				X
Quality of life - EQ-5D-5L (EuroQol		X				X
Self-reported use of healthcare		X				X
Adverse events				X	X	X
Self-reported use of smoking		X				X
INTERVENTIONS						
Smoking Cessation Intervention			X			
Referral to smoking cessation service			X			
E-cigarette given			X			
Signposting to NHS smoking			X			

Randomisation

Participants are individually randomised to intervention or control (1:1 ratio). Randomisation is by automated web-based system, computer generated by the UKCRC-registered Norwich Clinical Trials Unit (NCTU) using a blocked design, stratified by site.

For peer review only - http://bmjopen.bmj.com/site/about/guidelines.xhtml

Blinding

Due to the participatory nature of the intervention, it is impossible to blind participants or treating advisors to group allocation. Members of the trial management team will not be blinded to group allocation when assessing summary statistics for monitoring purposes throughout the trial. Analysis of the primary and secondary outcomes will be undertaken by a statistician blind to group allocation.

Internal pilot

The Independent Data Monitoring Committee (DMC) and Independent Trial Steering Committee (TSC) will scrutinise recruitment and protocol fidelity at three months into recruitment to establish continuation or stopping the trial at the pilot stage.

Follow up

1 Month and 3 Month Follow-Up

At one- and three-months post-randomisation, text messages are automatically generated by the study database and sent to all trial participants to ask about smoking status and any hospital admissions (to capture potential serious adverse events). If after two attempts there is no response, the central trial team follow-up by email, telephone, text or post.

6 Month Follow-up

At six months post randomisation, a link is automatically generated by the database and sent by text/email (depending on participant preference). The questionnaires consist of smoking status, time to relapse (if applicable), nicotine product use, e-cigarette use, use of healthcare and smoking cessation services in the past six months, self-reported dry cough and throat irritation, the Fagerstrom test for nicotine dependence (17), the Motivation to Stop Smoking scale (MTSS) (18), quality of life (EQ-5D-5L) (19) and health service use (which captures potential serious adverse events). If participants do not respond the central trial team follows-up by email, telephone, text, or post.

For those who report smoking abstinence at six months, biochemical validation is requested by CO breath test by three potential mechanisms. Participants can either [1] return to the hospital they attended at baseline, [2] undertake remote collection with a CO monitor that is sent to them (an appointment will be made with the central or local team for a video-call to retrieve the reading), [3] be visited at their home by a member of the local or central research team, or [4] meet with a member of the local or central team at a mutually acceptable place. On completing six month follow-up (including CO verification) randomised participants are reimbursed for their time with a £30 shopping voucher.

Trial allocation groups

Control

All trial participants are offered signposting to local NHS stop-smoking services through provision of written information. In the control arm this information is provided to the participant by the member of the research team taking consent; for the intervention group it forms part of the written information provided as part of the intervention.

Intervention

For peer review only - http://bmjopen.bmj.com/site/about/guidelines.xhtml

Those randomised to the intervention receive the CoSTED intervention, which is an opportunistic smoking cessation intervention undertaken in the ED, comprising three elements: 1) brief theory driven ((20)smoking cessation advice (up to 15 minutes), 2) the provision of an e-cigarette plus instruction in its use (up to 15 minutes), 3) referral to local stop-smoking services.

Following trial specific intervention training, the advice will be delivered individually (or with an accompanying person) by a smoking cessation advisor. Protocol driven advice addresses key aspects of the importance of switching away from tobacco smoking, tailored to the participant (e.g., discussing improved wound healing for patients attending with an open wound, or discussing increased risk of fractures for those who continue to smoke, for those attending with a fracture). This part of the intervention is a single session undertaken within the ED department, in a quiet area or in a separate room if available. Participants are provided with an e-cigarette starter kit (the DotPro starter kit, manufactured by Liberty Flights, an independent e-cigarette manufacturer). which includes approximately two weeks supply of refill pods and training on its use. Participants are also offered referral to the local stop-smoking service who will provide routine follow up.

Those delivering the intervention, mainly nurses and health care assistants, undertook 2.5 days of standardised training and were provided with an intervention manual and videos . Training included online modules from the National Centre for Smoking Cessation (NCSCT) (NCSCT Training and Assessment Programme – core components, then speciality courses for certified practitioners, including modules on 'mental health and smoking cessation', 'pregnancy and smoking cessation', 'E-cigarettes: a guide for healthcare professionals', and 'very brief advice on second-hand smoke'). Following this, NCSCT Level 2 smoking cessation advisor training was delivered by SmokeFree Norfolk and bespoke training on use of e-cigarettes, which was initially delivered face-to-face and then video-recorded for future training. All those trained had the opportunity to undertake some role-play and/or shadow a trained advisor prior to delivering the intervention themselves. Methods to enhance and assess intervention fidelity are informed by the National Institutes of Health (NIH) Behaviour Change Consortium fidelity framework (21).

Sample size

The sample size was based on an estimated quit rate of 12.2% in the intervention group. This was based on a US trial of an ED smoking cessation intervention using a brief intervention, referral to smoking cessation services and nicotine replacement (22). A quit rate of 6.2% was used in the control group based on an average of three studies of unmotivated quitters who received either signposting or no intervention (23–25). This is also similar to the results of a recent Cochrane review of incentives reporting a quit rate of 7.2% amongst controls (26). A sample size using 90% power gives a required sample size of 972 (486 per group) at the 5% level of significance using a two-tailed test. No increase for dropout has been made as dropouts will be assumed to have returned to smoking as is standard in the smoking cessation literature (27).

Outcome measures

See Table 1

Primary outcome

The primary effectiveness outcome is self-reported continuous smoking abstinence, biochemically validated by CO monitoring at six months with a cut off of ≥8ppm (i.e., a reading of ≤7 will denote abstinence), according to the Russell standard (27). If CO readings cannot be gathered, the participant is assumed to be smoking.

Other outcome measures

For peer review only - http://bmjopen.bmj.com/site/about/guidelines.xhtml

Secondary outcomes measured at 6 months from randomisation:

1. 7-day point prevalence abstinence (i.e., current smoking status, self-report of having smoked no cigarettes (not even a puff) in the past seven days, biochemically validated by carbon monoxide monitoring with cut off of ≥8 ppm)
2. Number of quit attempts
3. Time to relapse (if applicable)
4. Number of cigarettes per day
5. Nicotine dependence (17)
6. Number of times using an e-cigarette per day
7. Incidence of self-reported dry cough or mouth or throat irritation(10)
8. Motivation to Stop Smoking (25)
9. Self-reported use of healthcare services in the last 6 months
10. Self-reported use of smoking cessation services in last 6 months
11. Quality of life (using the EQ-5D-5L)(19)
12. Self-reported smoking status and adverse events/reactions will be collected at 1 and 3 months.

Economic evaluation

The economic evaluation takes the form of an incremental cost-effectiveness analysis (CEA) from an NHS /PSS perspective on an 'intention to treat' (ITT) basis following NICE guidance (28). The costs comprise intervention/usual care costs and wider health care service costs. Intervention costs are prospectively recorded alongside the intervention delivery. A self-completion service use questionnaire is used to collect participants' utilisation of wider health care services at baseline and six month follow-up. A set of national average unit costs from secondary sources will be applied to derive a health care cost profile for each patient. We also collect participants' out-of-pocket expenditure in relation to smoking cessation to capture potential financial burdens for participants.

The economic evaluation will use both smoking abstinence at six months post-randomisation and Quality Adjusted Life Years (QALYs), derived from EQ-5D-5L (19), as outcomes.

The primary economic analysis is a within-trial CEA using total costs over the trial period and smoking abstinence at six months follow-up. As the effects of smoking cessation on health and health care utilisation are likely to occur beyond the trial period, to demonstrate the effects beyond the time horizon of the trial we will undertake model-based lifetime projections to estimate the long-term cost-effectiveness of the intervention. To reflect the full impact of smoking cessation on health, we will also estimate lifetime incremental cost-effectiveness ratios (ICERs) using model-based projections.

Process evaluation

A mixed methods process evaluation (26) will assess implementation and explore participant views on the intervention compared to usual care, contextual variation and potential contamination between the intervention and control group.

Participant interviews

Detailed qualitative data will be collected through semi-structured interviews with a purposive sample of both intervention and control group participants (total n=30) after completion of final

For peer review only - http://bmjopen.bmj.com/site/about/guidelines.xhtml

follow-up. Participants will have been given the option at baseline to consent to being contacted about the qualitative interviews and formal consent will be sought.

Interviews will be undertaken face-to-face or remotely via telephone or video-conferencing and audio-recorded for transcription. The topic guide for intervention participants will enquire about views and experiences of the intervention, barriers and facilitators, and explore their longer-term perspective beyond the intervention period. For control participants the topic guide will explore experience of trial participation and access to usual care smoking cessation support, or any other form of support accessed.

Smoking cessation advisor interviews

At least one smoking cessation advisor from each of the six ED sites delivering the intervention will take part in a qualitative interview on completion of recruitment. Staff interviews will assess views and experiences of intervention delivery, giving an insider perspective on which parts of the intervention package are deemed to be most helpful, in which circumstances and with which participants. Barriers and facilitators to intervention delivery will be explored from the staff perspective, to aid interpretation of trial findings and triangulate with participants' qualitative data.

Interview data will be analysed thematically (29) , using a combination of inductive and deductive approaches to assess process elements of the trial. Initially all data will be analysed deductively, guided by the MRC guidance for complex interventions (30,31). Data will then be analysed inductively and more broadly. This will include critiquing the conceptual approach of CoSTED, guided by its logic model understanding any unintended consequences and reflections on the intervention from the participants' perspectives.

Site observations

Observational data of the intervention delivery and ED setting will be collected at each of the six ED sites. A researcher will attend for at least three hours and take notes about the context, the interactions and conversation between patients approached for the trial and staff involved. A structured observation record sheet will be used for this purpose, prompting the researcher to observe the environment, interactions between key actors, the culture of the department, the flow of patients and any critical events that may impact on intervention delivery and implementation. The researcher will also broadly document details of the setting, including any smoking and vaping observed in and around the department. This will aid in assessing context of intervention delivery. A Site Profile Form, completed prior to site observations as part of the Site Initiation Visit (SIV), will capture information that will support the researcher observations. Observation notes will be coded thematically using QSR NVivo and triangulated with interview data.

Descriptive quantitative measures: fidelity, dose, and reach

Quantitative process measures such as recruitment and consent rates, retention and drop out, will be analysed to help provide an explanatory context for eventual trial outcomes. The smoking cessation advisor will complete a brief feedback form after each intervention contact to enable monitoring of fidelity (e.g., incorporation of motivational advice and device demonstration) and 'dose' (e.g., duration of consultation, interruptions to intervention delivery). The reach of the intervention will be evaluated using socio-demographic data collected for all participants.

For peer review only - http://bmjopen.bmj.com/site/about/guidelines.xhtml

Trial data handling

Data are collected and maintained in accordance with the current legal and regulatory requirements.

The REDCap database and associated code have been developed by NCTU Data Management, in conjunction with the CoSTED trial team. The database software provides a number of features to help maintain data quality, including maintaining an audit trail, allowing custom validations on all data, allowing users to raise data query requests, and search facilities to identify validation failure/ missing data.

After completion of the trial, the database will be retained on the servers of NCTU for on-going analysis of secondary outcomes and held for five years in line with our data retention policy.

The identification, screening and enrolment logs, linking participant identifiable data to the pseudo-anonymised PIN, will be held centrally in REDCap to enable us to monitor screening progress and send out the SMS messages and emails. Screening forms will either be held in written form in a locked filing cabinet or electronically in a password protected form on hospital computers. After completion of the trial the identification, screening and enrolment logs will be stored securely by the sites for five years.

Qualitative interview data will be collected on an audio recorder, or by using computer recording via video conference, and immediately transferred to secure cloud storage (NCTU Sharepoint) with access restricted to nominated members of the research team. Audio files will be transcribed verbatim by a member of UEA administrative staff or by an external transcription company. When transcribed, a researcher will assign each transcript a unique study specific number and/or code and remove all identifying information from the transcripts. The anonymised data will be used for analysis and stored securely for five years unless otherwise advised by NCTU.

Observational notes will also be assigned a unique identifying code and stored securely at UEA with access restricted to nominated members of the research team.

Data analysis

A full statistical analysis plan was drafted during the trial delivery phase and will be reviewed and signed off by the independent DMC and TSC prior to database lock. The analyses will be reported in full and in accordance with the Consolidated Standards of Reporting Trials (CONSORT) guidelines (32). The main planned analyses are summarised below.

The comparison of outcomes between the treatment groups will be based on the ITT population. This will consist of all randomised individuals according to the group that they were allocated. For individuals who have missing data for the primary outcome it will be assumed that they are smoking. The primary outcome measure, smoking abstinence, will be compared between treatment groups using a binomial regression model adjusting for the factors stratified in the randomisation and any baseline factors that will be pre-specified by the Trial Management Group (TMG) prior to database lock, and the decision will be made blind to treatment allocation. The effect size will be estimated as the relative risk (log link function in the binomial regression) and the risk difference (identity link function).

For peer review only - http://bmjopen.bmj.com/site/about/guidelines.xhtml

Secondary outcome measures will be analysed in a similar fashion, but the regression model will depend upon the outcome, specifically, binomial regression for binary outcomes, Poisson/Negative binomial for count outcomes and linear regression for continuous outcomes. If appropriate non-parametric methods will be used.

The pattern of missing data will be assessed, and multiple imputation methods will be used to assess for sensitivity of the results to assumptions around the missing data.

DISCUSSION

This pragmatic RCT aims to determine the effectiveness of an opportunistic smoking cessation intervention (provision of an e-cigarette, brief advice, and referral for stop smoking support) delivered to participants whilst in an Emergency Department. This trial is unique in that smoking cessation interventions in UK EDs have never, to our knowledge, been tested in a trial. This trial will provide valuable insights into a novel location for prompting positive behaviour change.

Current study status

The CoSTED study started participant recruitment in January 2022. Data collection for the 6 month follow ups is expected to be completed in April 2023 and results are expected to be published in late 2023.

ETHICS AND DISSEMINATION:

The trial was approved by the South Central – Oxford B Research Committee (21/SC/0288). Dissemination will include the publication of outcomes, and the process and economic evaluations in peer reviewed journals. The findings will also be appropriately disseminated to relevant practice, policy and patient representative groups.

Acknowledgements We appreciate the support of the study sponsor, Norfolk and Norwich University Hospital NHS Foundation Trust (Norfolk and Norwich University Hospital Colney Lane Norwich NR4 7UY Tel: 01603 286286). The study was led by researchers at the University of East Anglia (UEA) and managed by the Norwich Clinical Trials Unit (NCTU) at UEA. Our thanks go to all organisations involved in recruitment and in supporting delivery of the intervention, particularly local stop-smoking services. We would also like to thank PPI contributors who were critical in defining choice of e cigarette starter kit and shaping the intervention. We acknowledge the contribution of the researchers at sites who underwent training to deliver the intervention. We thank SmokeFree Norfolk for supporting the study by training all smoking advisors. Thanks also to our independent Trial Steering Committee and Data Monitoring committee members.

Contributors statement IP and CN conceived the idea for the study.CN, IP, EW and PB developed the intervention. LC is the trial manager and MM is the Senior Trial Manager. AC is the study statistician, SP is the Health Economist. SA, BB, AB, GM, AG, TC are site PIs, LB and RH provide academic expertise. All authors contributed to protocol writing and approved the final version.

Competing interests – None

Funding This study is funded by the National Institute for Health Research (NIHR) HTA programme Tobacco Cessation, Control and Harm Reduction (project reference 129438).

For peer review only - http://bmjopen.bmj.com/site/about/guidelines.xhtml

Disclaimer Neither the study sponsor or funder has a direct role in the study design; data collection, management, analysis and interpretation of data, report/manuscript writing or results dissemination. The views expressed are those of the author(s) and not necessarily those of the NIHR or the Department of Health and Social Care.

Data sharing statement Data underlying the main trial results will be available in a public, open access repository immediately following publication of the major findings of the study. Details of the repository and a persistent URL will be provided on publication of the main results. Study protocol, statistical analysis plan, informed consent form, and other documents will also be available.

REFERENCES

1. Chan SSC, Leung DYP, Abdullah ASM, Wong VT, Hedley AJ, Lam TH. A randomized controlled trial of a smoking reduction plus nicotine replacement therapy intervention for smokers not willing to quit smoking. Addiction. 2011 Jun 1;106(6):1155–63.

2. Adult smoking habits in the UK - Office for National Statistics [Internet]. [cited 2019 Aug 7]. Available from: https://www.ons.gov.uk/peoplepopulationandcommunity/healthandsocialcare/healthandlifeexpectancies/bulletins/adultsmokinghabitsingreatbritain/2017#the-proportion-who-are-current-smokers-in-the-uk-its-constituent-countries-and-local-areas-2011-to-2017

3. Royal College of Physicians. Hiding in plain sight. Treating tobacco dependency in the NHS. A report by the Tobacco Advisory Group of the Royal College of Physicians. 2018.

4. Nicotine without smoke: Tobacco harm reduction [Internet]. RCP London. 2016 [cited 2018 Jul 31]. Available from: https://www.rcplondon.ac.uk/projects/outputs/nicotine-without-smoke-tobacco-harm-reduction-0

5. Keene J, Rodriguez J. Are mental health problems associated with use of Accident and Emergency and health-related harm? Eur J Public Health. 2007 Aug 1;17(4):387–93.

6. Latest Statistics - Smoking In England [Internet]. [cited 2020 Feb 3]. Available from: http://www.smokinginengland.info/latest-statistics/

7. Boudreaux ED, Baumann BM, Friedman K, Ziedonis DM. Smoking Stage of Change and Interest in an Emergency Department–based Intervention. Academic Emergency Medicine. 2005;12(3):211–8.

8. Drummond C, Deluca P, Coulton S, Bland M, Cassidy P, Crawford M, et al. The effectiveness of alcohol screening and brief intervention in emergency departments: a multicentre pragmatic cluster randomized controlled trial. PLoS One. 2014;9(6):e99463.

9. Lemhoefer C, Rabe GL, Wellmann J, Bernstein SL, Cheung KW, McCarthy WJ, et al. Emergency Department-Initiated Tobacco Control: Update of a Systematic Review and Meta-Analysis of Randomized Controlled Trials. Prev Chronic Dis. 2017;14:E89–E89.

10. Hajek P, Phillips-Waller A, Przulj D, Pesola F, Myers Smith K, Bisal N, et al. A Randomized Trial of E-Cigarettes versus Nicotine-Replacement Therapy. N Engl J Med. 2019 Jan 30;380(7):629–37.

For peer review only - http://bmjopen.bmj.com/site/about/guidelines.xhtml

11. Hartmann-Boyce J, McRobbie H, Butler AR, Lindson N, Bullen C, Begh R, et al. Electronic cigarettes for smoking cessation. Cochrane Database of Systematic Reviews [Internet]. 2021 [cited 2021 Jul 27];(4). Available from: https://www.cochranelibrary.com/cdsr/doi/10.1002/14651858.CD010216.pub5/full

12. English TM, Smith TB, Song X, Whitman MV. Barriers to electronic cigarette use. Public Health Nursing. 2018;35(5):363–8.

13. Department of Health and Social Care. Prevention is better than cure [Internet]. Available from: https://assets.publishing.service.gov.uk/government/uploads/system/uploads/attachment_data/file/753688/Prevention_is_better_than_cure_5-11.pdf

14. Health A on S and. Smoking Still Kills: Protecting Children, Reducing Inequalities. London: ASH; 2015.

15. Chan AW, Tetzlaff JM, Altman DG, Laupacis A, Gøtzsche PC, Krleža-Jerić K, et al. SPIRIT 2013 statement: defining standard protocol items for clinical trials. Ann Intern Med. 2013 Feb 5;158(3):200–7.

16. Hoffmann TC, Glasziou PP, Boutron I, Milne R, Perera R, Moher D, et al. Better reporting of interventions: template for intervention description and replication (TIDieR) checklist and guide. BMJ. 2014 Mar 7;348:g1687.

17. Heatherton TF, Kozlowski LT, Frecker RC, Fagerstrom K olov. The Fagerstrom Test for Nicotine Dependence: a revision of the Fagerstrom Tolerance Questionnaire. Br J Addict. 1991;1119–27.

18. Crittenden KS, Manfredi C, Lacey L, Warnecke R, Parsons J. Measuring readiness and motivation to quit smoking among women in public health clinics. Addictive Behaviors. 1994 Sep 1;19(5):497–507.

19. EQ-5D-5L – EQ-5D [Internet]. [cited 2020 Nov 8]. Available from: https://euroqol.org/eq-5d-instruments/eq-5d-5l-about/

20. Michie S, van Stralen MM, West R. The behaviour change wheel: A new method for characterising and designing behaviour change interventions. Implement Sci. 2011 Apr 23;6:42.

21. Borrelli B. The assessment, monitoring, and enhancement of treatment fidelity in public health clinical trials. J Public Health Dent. 2011;71 Suppl 1:S52-63.

22. Bernstein SL, D'Onofrio G, Rosner J, O'Malley S, Makuch R, Busch S, et al. Successful Tobacco Dependence Treatment in Low-Income Emergency Department Patients: A Randomized Trial. Annals of Emergency Medicine. 2015 Aug;66(2):140–7.

23. Hennrikus D, Joseph AM, Lando HA. Effectiveness of a Smoking Cessation Program for Peripheral Artery Disease Patients: A Randomized Controlled Trial. Journal of Vascular Surgery. 2011 Jun 1;53(6):1752.

24. McFall M, Saxon AJ, Malte CA, Chow B, Bailey S, Baker DG, et al. Integrating Tobacco Cessation Into Mental Health Care for Posttraumatic Stress Disorder: A Randomized Controlled Trial. JAMA. 2010 Dec 8;304(22):2485–93.

For peer review only

25. Pieterse ME, Seydel ER, DeVries H, Mudde AN, Kok GJ. Effectiveness of a minimal contact smoking cessation program for Dutch general practitioners: a randomized controlled trial. Prev Med. 2001 Feb;32(2):182–90.

26. Notley C, Gentry S, Livingstone-Banks J, Bauld L, Perera R, Hartmann-Boyce J. Incentives for smoking cessation. Cochrane Database of Systematic Reviews [Internet]. 2019 [cited 2019 Aug 7];(7). Available from: https://www.cochranelibrary.com/cdsr/doi/10.1002/14651858.CD004307.pub6/full

27. West R. Assessing smoking cessation performance in NHS Stop Smoking Services: The Russell Standard (Clinical). 2005.

28. 1 Introduction | Developing NICE guidelines: the manual | Guidance | NICE [Internet]. NICE; [cited 2022 Apr 7]. Available from: https://www.nice.org.uk/process/pmg20/chapter/introduction

29. Braun V, Clarke V. Using thematic analysis in psychology. Qualitative Research in Psychology. 2006 Jan 1;3(2):77–101.

30. Moore GF, Audrey S, Barker M, Bond L, Bonell C, Hardeman W, et al. Process evaluation of complex interventions: Medical Research Council guidance. BMJ. 2015 Mar 19;350:h1258.

31. Craig P, Dieppe P, Macintyre S, Michie S, Nazareth I, Petticrew M. Developing and evaluating complex interventions: the new Medical Research Council guidance. BMJ [Internet]. 2008 Sep 29;337. Available from: https://www.ncbi.nlm.nih.gov/pmc/articles/PMC2769032/

32. Tong A, Sainsbury P, Craig J. Consolidated criteria for reporting qualitative research (COREQ): a 32-item checklist for interviews and focus groups. Int J Qual Health Care. 2007 Dec 1;19(6):349–57.

Figure legend:

Figure 1: Participant Flow Diagram

For peer review only

For peer review only - http://bmjopen.bmj.com/site/about/guidelines.xhtml

Figure 1: Participant Flow Diagram

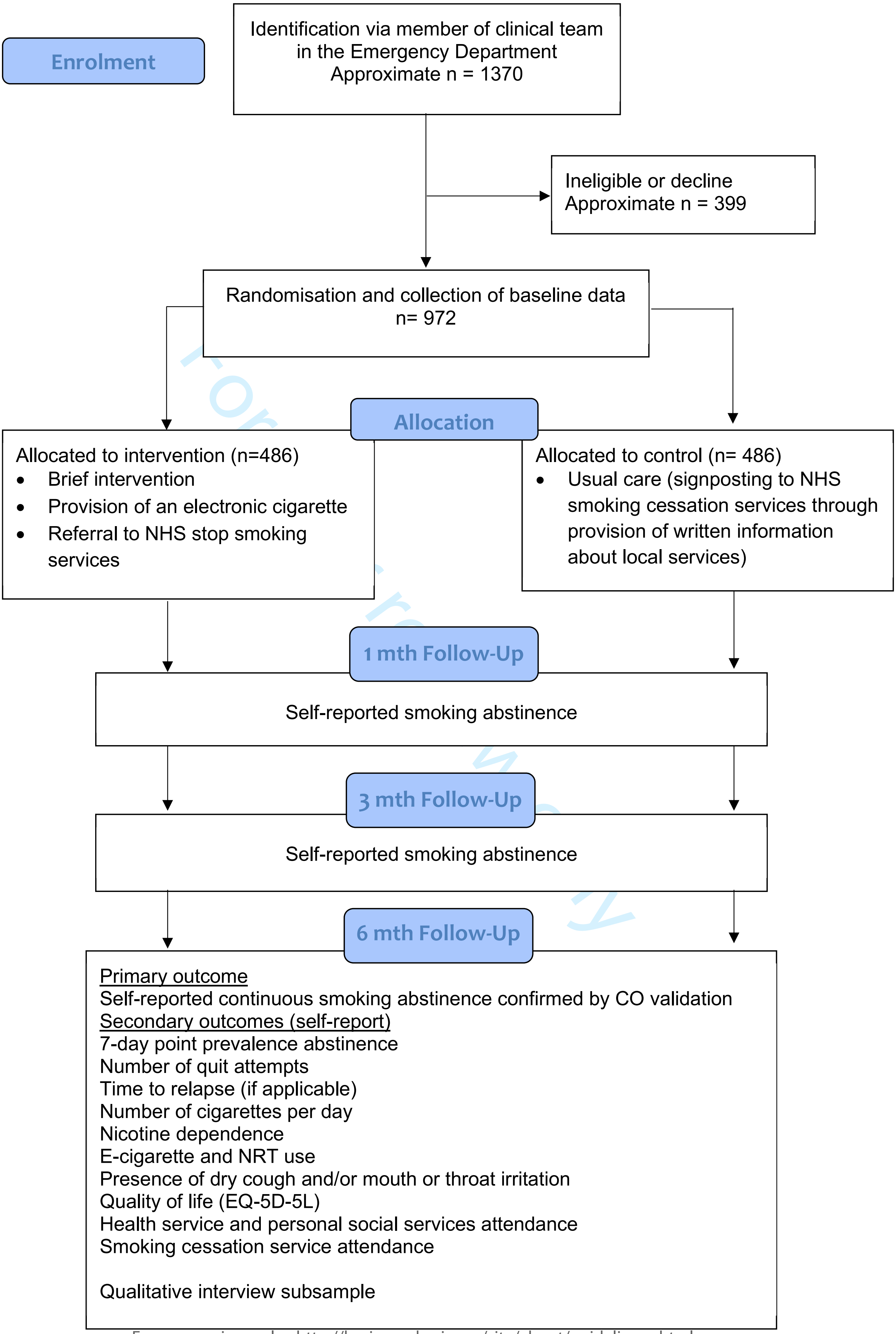

For peer review only

SPIRIT

STANDARD PROTOCOL ITEMS: RECOMMENDATIONS FOR INTERVENTIONAL TRIALS

SPIRIT 2013 Checklist: Recommended items to address in a clinical trial protocol and related documents*

Section/item	Item No	Description
**Administrative information**		
Title	1	Descriptive title identifying the study design, population, interventions, and, if applicable, trial acronym. Page 1.
Trial registration	2a	Trial identifier and registry name. If not yet registered, name of intended registry. Page 2.
	2b	All items from the World Health Organization Trial Registration Data Set N/A
Protocol version	3	Date and version identifier. Page 2.
Funding	4	Sources and types of financial, material, and other support. Page 12.
Roles and responsibilities	5a	Names, affiliations, and roles of protocol contributors. Page 1.
	5b	Name and contact information for the trial sponsor. Page 12.
	5c	Role of study sponsor and funders, if any, in study design; collection, management, analysis, and interpretation of data; writing of the report; and the decision to submit the report for publication, including whether they will have ultimate authority over any of these activities. Page 13.
	5d	Composition, roles, and responsibilities of the coordinating centre, steering committee, endpoint adjudication committee, data management team, and other individuals or groups overseeing the trial, if applicable (see Item 21a for data monitoring committee). Open Science framework.
**Introduction**		
Background and rationale	6a	Description of research question and justification for undertaking the trial, including summary of relevant studies (published and unpublished) examining benefits and harms for each intervention. Page 3
	6b	Explanation for choice of comparators. Page 7
Objectives	7	Specific objectives or hypotheses. Page 3-4

For peer review only

Trial design	8	Description of trial design including type of trial (eg, parallel group, crossover, factorial, single group), allocation ratio, and framework (eg, superiority, equivalence, noninferiority, exploratory). Pge 5.

Methods: Participants, interventions, and outcomes

Study setting	9	Description of study settings (eg, community clinic, academic hospital) and list of countries where data will be collected. Reference to where list of study sites can be obtained. Page 5.
Eligibility criteria	10	Inclusion and exclusion criteria for participants. If applicable, eligibility criteria for study centres and individuals who will perform the interventions (eg, surgeons, psychotherapists). Page 5.
Interventions	11a	Interventions for each group with sufficient detail to allow replication, including how and when they will be administered. Page 7-8
	11b	Criteria for discontinuing or modifying allocated interventions for a given trial participant (eg, drug dose change in response to harms, participant request, or improving/worsening disease) N/A as intervention delivered immediately following baseline assessment
	11c	Strategies to improve adherence to intervention protocols, and any procedures for monitoring adherence (eg, drug tablet return, laboratory tests) Page 10
	11d	Relevant concomitant care and interventions that are permitted or prohibited during the trial. N/A.
Outcomes	12	Primary, secondary, and other outcomes, including the specific measurement variable (eg, systolic blood pressure), analysis metric (eg, change from baseline, final value, time to event), method of aggregation (eg, median, proportion), and time point for each outcome. Explanation of the clinical relevance of chosen efficacy and harm outcomes is strongly recommended. Page 8 & Page 6 (table 1)
Participant timeline	13	Time schedule of enrolment, interventions (including any run-ins and washouts), assessments, and visits for participants. A schematic diagram is highly recommended (see Figure). Page 7.
Sample size	14	Estimated number of participants needed to achieve study objectives and how it was determined, including clinical and statistical assumptions supporting any sample size calculations. Page 8.
Recruitment	15	Strategies for achieving adequate participant enrolment to reach target sample size. Page 5/6.

Methods: Assignment of interventions (for controlled trials)

Allocation:

Sequence generation	16a	Method of generating the allocation sequence (eg, computer-generated random numbers), and list of any factors for stratification. To reduce predictability of a random sequence, details of any planned restriction (eg, blocking) should be provided in a separate document that is unavailable to those who enrol participants or assign interventions. Page 6.
Allocation concealment mechanism	16b	Mechanism of implementing the allocation sequence (eg, central telephone; sequentially numbered, opaque, sealed envelopes), describing any steps to conceal the sequence until interventions are assigned. Page 6.
Implementation	16c	Who will generate the allocation sequence, who will enrol participants, and who will assign participants to interventions. Page 6.
Blinding (masking)	17a	Who will be blinded after assignment to interventions (eg, trial participants, care providers, outcome assessors, data analysts), and how. Page 7.
	17b	If blinded, circumstances under which unblinding is permissible, and procedure for revealing a participant's allocated intervention during the trial. Page 7.
**Methods: Data collection, management, and analysis**		
Data collection methods	18a	Plans for assessment and collection of outcome, baseline, and other trial data, including any related processes to promote data quality (eg, duplicate measurements, training of assessors) and a description of study instruments (eg, questionnaires, laboratory tests) along with their reliability and validity, if known. Reference to where data collection forms can be found, if not in the protocol. Page 7.
	18b	Plans to promote participant retention and complete follow-up, including list of any outcome data to be collected for participants who discontinue or deviate from intervention protocols. Page 7.
Data management	19	Plans for data entry, coding, security, and storage, including any related processes to promote data quality (eg, double data entry; range checks for data values). Reference to where details of data management procedures can be found, if not in the protocol. Data sharing plan.
Statistical methods	20a	Statistical methods for analysing primary and secondary outcomes. Reference to where other details of the statistical analysis plan can be found, if not in the protocol. Page 11 & SAP.
	20b	Methods for any additional analyses (eg, subgroup and adjusted analyses). Page 11 & SAP.
	20c	Definition of analysis population relating to protocol non-adherence (eg, as randomised analysis), and any statistical methods to handle missing data (eg, multiple imputation). Page 11 & SAP.

For peer review only - http://bmjopen.bmj.com/site/about/guidelines.xhtml

**Methods: Monitoring**		
Data monitoring	21a	Composition of data monitoring committee (DMC); summary of its role and reporting structure; statement of whether it is independent from the sponsor and competing interests; and reference to where further details about its charter can be found, if not in the protocol. Alternatively, an explanation of why a DMC is not needed. Open Science framework
	21b	Description of any interim analyses and stopping guidelines, including who will have access to these interim results and make the final decision to terminate the trial. Page 11 & SAP.
Harms	22	Plans for collecting, assessing, reporting, and managing solicited and spontaneously reported adverse events and other unintended effects of trial interventions or trial conduct. Page 7 and page 6 (table 1)
Auditing	23	Frequency and procedures for auditing trial conduct, if any, and whether the process will be independent from investigators and the sponsor. According to sponsor / funder request.
**Ethics and dissemination**		
Research ethics approval	24	Plans for seeking research ethics committee/institutional review board (REC/IRB) approval. Page 12.
Protocol amendments	25	Plans for communicating important protocol modifications (eg, changes to eligibility criteria, outcomes, analyses) to relevant parties (eg, investigators, REC/IRBs, trial participants, trial registries, journals, regulators). Page 12,
Consent or assent	26a	Who will obtain informed consent or assent from potential trial participants or authorised surrogates, and how (see Item 32). Page 5/6
	26b	Additional consent provisions for collection and use of participant data and biological specimens in ancillary studies, if applicable. N/A.
Confidentiality	27	How personal information about potential and enrolled participants will be collected, shared, and maintained in order to protect confidentiality before, during, and after the trial. Page 11.
Declaration of interests	28	Financial and other competing interests for principal investigators for the overall trial and each study site. Page 12.
Access to data	29	Statement of who will have access to the final trial dataset, and disclosure of contractual agreements that limit such access for investigators. Page 11 and data management plan,
Ancillary and post-trial care	30	Provisions, if any, for ancillary and post-trial care, and for compensation to those who suffer harm from trial participation. N/A.

For peer review only - http://bmjopen.bmj.com/site/about/guidelines.xhtml

Dissemination policy	31a	Plans for investigators and sponsor to communicate trial results to participants, healthcare professionals, the public, and other relevant groups (eg, via publication, reporting in results databases, or other data sharing arrangements), including any publication restrictions. Page 12.
	31b	Authorship eligibility guidelines and any intended use of professional writers. N/A.
	31c	Plans, if any, for granting public access to the full protocol, participant-level dataset, and statistical code. Page 13.
**Appendices**		
Informed consent materials	32	Model consent form and other related documentation given to participants and authorised surrogates. Page 13 (to be made available on open science framework)
Biological specimens	33	Plans for collection, laboratory evaluation, and storage of biological specimens for genetic or molecular analysis in the current trial and for future use in ancillary studies, if applicable. N/A.

*It is strongly recommended that this checklist be read in conjunction with the SPIRIT 2013 Explanation & Elaboration for important clarification on the items. Amendments to the protocol should be tracked and dated. The SPIRIT checklist is copyrighted by the SPIRIT Group under the Creative Commons "Attribution-NonCommercial-NoDerivs 3.0 Unported" license.

For peer review only

For peer review only - http://bmjopen.bmj.com/site/about/guidelines.xhtml

TIDieR
Template for Intervention Description and Replication

The TIDieR (Template for Intervention Description and Replication) Checklist*:

Information to include when describing an intervention and the location of the information

Item number	Item	Where located ** Primary paper (page or appendix number)	Other † (details)
	**BRIEF NAME**		
**1.**	Provide the name or a phrase that describes the intervention.	__N/A_______	_______________
	**WHY**		
**2.**	Describe any rationale, theory, or goal of the elements essential to the intervention.	___page 3___	______________
	**WHAT**		
**3.**	Materials: Describe any physical or informational materials used in the intervention, including those provided to participants or used in intervention delivery or in training of intervention providers. Provide information on where the materials can be accessed (e.g. online appendix, URL).	_____________	______________
**4.**	Procedures: Describe each of the procedures, activities, and/or processes used in the intervention, including any enabling or support activities.	______	______________
	**WHO PROVIDED**		
**5.**	For each category of intervention provider (e.g. psychologist, nursing assistant), describe their expertise, background and any specific training given.	__page 7____	______________
	**HOW**		
**6.**	Describe the modes of delivery (e.g. face-to-face or by some other mechanism, such as internet or telephone) of the intervention and whether it was provided individually or in a group.	__page 7_____	______________
	**WHERE**		
**7.**	Describe the type(s) of location(s) where the intervention occurred, including any necessary infrastructure or relevant features.	___page 7___	______________

For peer review only - http://bmjopen.bmj.com/site/about/guidelines.xhtml

	**WHEN and HOW MUCH**		
**8.**	Describe the number of times the intervention was delivered and over what period of time including the number of sessions, their schedule, and their duration, intensity or dose.	__page 7____	______________
	**TAILORING**		
**9.**	If the intervention was planned to be personalised, titrated or adapted, then describe what, why, when, and how.	___page 7___	______________
	**MODIFICATIONS**		
**10.‡**	If the intervention was modified during the course of the study, describe the changes (what, why, when, and how).	_____N/A_______ ___	______________
	**HOW WELL**		
**11.**	Planned: If intervention adherence or fidelity was assessed, describe how and by whom, and if any strategies were used to maintain or improve fidelity, describe them.	__ page 7 but not needed until outcome	______________
**12.‡**	Actual: If intervention adherence or fidelity was assessed, describe the extent to which the intervention was delivered as planned.	___N/A_______ ___	______________

** **Authors** - use N/A if an item is not applicable for the intervention being described. **Reviewers** – use '?' if information about the element is not reported/not sufficiently reported.

† If the information is not provided in the primary paper, give details of where this information is available. This may include locations such as a published protocol or other published papers (provide citation details) or a website (provide the URL).
‡ If completing the TIDieR checklist for a protocol, these items are not relevant to the protocol and cannot be described until the study is complete.

* We strongly recommend using this checklist in conjunction with the TIDieR guide (see *BMJ* 2014;348:g1687) which contains an explanation and elaboration for each item.

* The focus of TIDieR is on reporting details of the intervention elements (and where relevant, comparison elements) of a study. Other elements and methodological features of studies are covered by other reporting statements and checklists and have not been duplicated as part of the TIDieR checklist. When a **randomised trial** is being reported, the TIDieR checklist should be used in conjunction with the CONSORT statement (see www.consort-statement.org) as an extension of **Item 5 of the CONSORT 2010 Statement.** When a **clinical trial protocol** is being reported, the TIDieR checklist should be used in conjunction with the SPIRIT statement as an extension of **Item 11 of the SPIRIT 2013**

For peer review only - http://bmjopen.bmj.com/site/about/guidelines.xhtml

Statement (see www.spirit-statement.org). For alternate study designs, TIDieR can be used in conjunction with the appropriate checklist for that study design (see www.equator-network.org).

For peer review only

For peer review only - http://bmjopen.bmj.com/site/about/guidelines.xhtml
